# Overexpression of BRINP3 Predicts Poor Prognosis and Promotes Cancer Cell Proliferation and Migration via MAP4 in Osteosarcoma

**DOI:** 10.1155/2022/2698869

**Published:** 2022-07-07

**Authors:** Wenchao Zeng, Huiya Xu, Tian Wei, Huishan Liang, Xiaokun Ma, Fen Wang

**Affiliations:** ^1^Department of Hand Foot and Ankle Surgery, Jining No.1 People's Hospital, Ji Ning, 2720000, China; ^2^Department of Pathology, The First Affiliated Hospital, Sun Yat-sen University, Guangzhou 510080, China; ^3^Department of Medical Oncology, The Third Affiliated Hospital of Sun Yat-sen University, Guangzhou 510630, China

## Abstract

Osteosarcoma (OS) is a primary malignant bone tumor most commonly affecting children and adolescents and is characterized by loss of differentiation. Bone morphogenetic protein/retinoic acid inducible neural-specific 3 (BRINP3) has been reported to regulate the differentiation of osteoblasts. However, the role that BRINP3 plays in the progression of osteosarcoma remains unknown. We found in this study that BRINP3 was highly expressed in 64.13% of human osteosarcoma tissues and it was associated with histological grade, tumor recurrence, and poor clinical prognosis of osteosarcoma. In vitro, downregulation of BRINP3 was able to inhibit the proliferation and invasion of osteosarcoma cell lines. Furthermore, BRINP3 interacted with microtubule-associated protein 4 (MAP4) at the protein level, and overexpression of MAP4 could partially reverse the inhibitory effect of downregulated BRINP3 on the proliferation and invasion of osteosarcoma cells, which indicates that downregulation of BRINP3 might suppress the proliferation and invasion of osteosarcoma cells by inhibiting MAP4 expression. Overall, our results demonstrate that BRINP3 functions as an oncogene within osteosarcoma through MAP4 and could therefore be used as a potential biomarker for osteosarcoma diagnostics and therapeutics.

## 1. Introduction

Osteosarcoma (OS) is a primary malignant bone tumor most commonly found in children and adolescents. The number of osteosarcoma cases reported each year is approximately 4.4 per million children [1, 2]. Osteosarcoma is characterized by induced mesenchymal cells and bone destruction [3]. Although treatment strategies such as adjuvant chemotherapy, radiotherapy, and wide local tumor excision have been rapidly advancing, the 5-year survival rate of osteosarcoma is still unsatisfactory [4–7]. The poor prognosis is mainly attributed to recurrence and metastasis, which result in a 5-year survival rate lower than 20% [8]. Thus, it is pressing to uncover the fundamental mechanisms to pinpoint novel diagnostic and therapeutic targets for osteosarcoma.

Over 80% of osteosarcoma cells typically exhibit poor differentiation or no differentiation, and this loss of differentiation is a prominent characteristic of osteosarcoma [9]. As reported, disruption of differentiation can lead to the development of osteosarcoma, while differentiation of osteoblast can promote cell cycle arrest and inhibit tumor proliferation [10]. Noteworthily, bone morphogenetic protein/retinoic acid inducible neural-specific 3 (BRINP3), formerly known as family with sequence similarity 5, member C (FAM5C), has been observed to regulate osteoblast differentiation in differentiated osteoblasts [11]. By reducing of endogenous BRINP3, the levels of bone differentiation-related factors such as osterix, alkaline phosphatase (ALP), and osteocalcin (OCN) mRNA were decreased, as well as type 1 collagen and *β*-catenin levels in differentiated osteoblastic MC3T3-E1 cells. Meanwhile, overexpression of BRINP3 significantly increased the expression of osterix, OCN, and ALP mRNA induced by bone morphogenetic protein-2 in the precursor cells of osteoblast C2C12 cells. These results suggest that BRINP3 promotes bone differentiation in mature osteoblasts and inhibits bone differentiation in precursor osteoblasts (which might be similar to osteosarcoma cells). Nonetheless, the role of BRINP3 in the progression of osteosarcoma remains unknown.

The BRINP3 gene is reported to be correlated with the development of the nervous system [12, 13], myocardial infarction [14], aggressive periodontitis [15], and osteoblast differentiation [11] in genetic studies. Currently, the role the BRINP3 protein plays in the tumor is still not well understood. In their research on pituitary adenoma cells, BRINP3 was identified by Shorts-Cary et al. [16] as a mitochondrial-associated protein, which correlated with tumor proliferation, migration, and invasion. However, in tongue squamous cell carcinoma, FAM5C (BRINP3) has been found to act as a novel tumor suppressor gene [17]. Given the above different research results, the role that BRINP3 plays in osteosarcoma remains to be found out.

In this study, a high level of BRINP3 expression was observed in human osteosarcoma and BRINP3 was correlated with tissue differentiation and clinical prognosis of osteosarcoma. Specifically, BRINP3 downregulation could inhibit the proliferation and invasion of osteosarcoma cell lines. Through Co-IP and mass spectrometry protein identification, we found that the above function was performed at least partly through the downregulation of MAP4. As shown by our study results, BRINP3 might be utilized as a potential biomarker for diagnostic and therapeutic targets for osteosarcoma.

## 2. Materials and Methods

### 2.1. Patients and Specimens

Coarse needle puncture samples from 92 osteosarcoma patients were obtained before treatment from the First Affiliated Hospital of Sun Yat-sen University between 2010 and 2015, and the histopathology and follow-up information was completed. The tissue samples used for analysis were formalin-fixed and paraffin-embedded (FFPE). Institutional Review Board approval was obtained from the First Hospital of Sun Yat-sen University for this study. Human subjects were involved in all procedures according to the *Declaration of Helsinki*.

### 2.2. Immunohistochemistry

We cut paraffin-embedded samples into 4 *μ*m sections, and deparaffinized antigens were retrieved for 2 min in sodium citrate buffer (pH 6.0) at high temperature and pressure. After incubation with anti-BRINP3 (Signalway Antibody, Greenbelt, MD, USA) (1 : 400) overnight at 4°C, the slides were incubated with anti-mouse/rabbit IHC Secondary Antibody Kit (Gene Tech, Shanghai, China), and stained slides were scanned using a digital pathology slide scanner (KFBIO, Ningbo, China). Specimens were scored based on the intensity of staining and the positive area. The staining intensity was graded as 0 (negative), 1 (weak), 2 (moderate), and 3 (strong). In terms of positive area, the following areas were defined as 0 (≤5%), 1 (5-25%), 2 (26-50%), 3 (51-75%), and 4 (≥75%). The scores of the above two grades were added up, and specimens were divided into 4 levels based on the final scores: 0-1 scores (−), 2 scores (+), 3-4 scores (++), and more than 5 scores (+++). A score < 3 was defined as low BRINP3 expression, and a score ≥ 3 was defined as high BRINP3 expression.

### 2.3. Cell Lines and Cell Culture

The human osteosarcoma cell lines Saos-2, U2OS, MG-63, and HOS were obtained from the Cell Bank of Type Culture Collection of Chinese Academy of Sciences (Shanghai, China). And a short tandem repeat (STR) DNA profiling analysis was performed to verify. Cell culture was conducted in DMEM medium (Gibco, Gaithersburg, MD, USA) supplemented with 10% fetal bovine serum (Gibco), 100 *μ*g/ml streptomycin, and 100 *μ*g/ml penicillin. We maintained all cell lines at 37°C with 5% CO_2_ in a humidified incubator.

### 2.4. Transfection and Vector Construction

To generate the recombinant lentivirus, pGV115-BRINP3-shRNA plasmid or GV348-BRINP3 plasmid and packing plasmids pHelper 1.0 and pHelper 2.0 (Genechem, Shanghai, China) were cotransfected into 293 T cells for 6 h and then changed to normal media. Following 48 hours of transfection, lentivirus-containing culture medium was collected and concentrated.

### 2.5. Lentivirus-Based BRINP3 shRNA Silencing in Human Osteosarcoma Cells

U2OS cells and Saos-2 cells were transduced using the lentivirus plasmid pGV115-BRINP3-shRNA (Genechem) with 8 *μ*g/ml polybrene, and then, the expression of the green fluorescent label was observed. Cells were used for subsequent experiments, wherein lentiviral transfection efficiency exceeded 80%.

### 2.6. Establishment of Stable BRINP3-Overexpressed Cell Lines

We used the lentivirus plasmid pGV348-BRINP3 containing 3× Flag tag obtained from Genechem with 8 *μ*g/ml polybrene to infect U2OS cells and eventually screened out the stably overexpressed cell lines with puromycin (2 *μ*g/ml final concentration).

### 2.7. Real-Time PCR

The total amount of RNA extraction was performed by Trizol Reagent (SuPerfecTRI, Shanghai, China) and reversely transcribed using the M-MLV Reverse Transcriptase Kit (Promega, Madison, WI, USA). The RNA concentration was measured by NanoDrop. qPCR was performed in LightCycler 480 Real-Time PCR System (Roche, Basel, Switzerland) using SYBR Master Mixture (TAKARA, Otsu, Japan) following the manufacturer's instructions. We calculate the relative mRNA expression levels by using the 2^−*ΔΔ*CT^ method with GAPDH as the reference standard (Table S1).

### 2.8. Western Blot Assay

Using a BCA protein assay kit (Beyotime, Shanghai, China), we quantified the cellular proteins using RIPA lysis buffer (Beyotime) containing 1% phosphatase and protease inhibitors. After separation on SDS-PAGE, the proteins were transferred to PVDF membranes (Millipore, Billerica, MA, USA). Using 5% fat-free milk as a blocking agent at room temperature for 1 h, the membranes were incubated overnight with primary antibodies at 4°C. Subsequently, the membranes were washed with 1× TBST and incubated with secondary antibodies (1 : 2000) for 1 h at room temperature. The proteins were then developed using the enhanced chemiluminescence ECL reagent (Thermo Fisher Scientific, San Jose, CA, USA). Relative protein levels were determined with GAPDH (1 : 5000) as a reference. Detailed antibody staining information is provided in Table S2.

### 2.9. Cell Proliferation Analysis

Cell proliferation and viability were measured using the Celigo imaging cytometry system and MTT assay, respectively. Cells from different treatment groups were trypsinized in the logarithmic growth phase using trypsin (Sangon, Shanghai, China), resuspended in standard media, and seeded into 96-well plates (2 × 10^3^ cells/well).

The cell proliferation was quantified using a Celigo Image Cytometer (Nexcelom, Lawrence, MA, USA) by counting green fluorescence for 5 consecutive days. To measure cell viability, 20 *μ*l of 5 mg/ml MTT solution (Genview, El Monte, CA, USA) was added to each well and then the cells were incubated for another 4 h. Cell viability was assessed at 490/570 nm at various time points.

### 2.10. Invasion Assay

Matrigel Basement Membrane Matrix (Trevigen, Gaithersburg, MD, USA) was coated on the surface of 24-well BioCoat cell culture inserts (Costar, Corning, NY, USA) with 8-lm-porosity polyethylene terephthalate membranes to estimate the invasive ability of the cells.

### 2.11. Coimmunoprecipitation and Mass Spectrometry

To perform coimmunoprecipitation, we transfected the indicated constructs into cells for 48 hours and then treated them with IP lysis buffer (Beyotime) plus protease inhibitor cocktail (Beyotime) for 1 hour at 4°C. Following full-speed centrifugation for 10 min, the supernatant was gently rotated by using antibodies and protein A beads (Invitrogen, Carlsbad, CA, USA) for 4 h at 4°C. Afterward, the beads were washed three times with lysis buffer and centrifuged at 500 *g* for 5 min before collection. The beads were eluted in 1× SDS-PAGE loading buffer to obtain precipitated proteins. Subsequently, western blotting was performed using the precipitated proteins and cell lysates indicated above.

SDS-PAGE electrophoresis and Coomassie brilliant blue staining were performed on the coimmunoprecipitation samples. Then, the strips were cut off, and the protein in each sample strip was hydrolyzed by trypsin to cut the protein into peptide segments. The mass spectrometry analyses were performed using Easy-nLC 1000 system (Thermo Fisher Scientific) and Q-Exactive Plus mass spectrometer (Thermo Fisher Scientific).

### 2.12. Statistical Analysis

Data are shown as the mean ± standard deviation (SD). All data were analyzed using GraphPad Prism software 8.0 (GraphPad Software, San Diego, CA, USA). *P* < 0.05 indicated a statistically significant difference.

## 3. Results

### 3.1. BRINP3 Is Upregulated in Clinical Osteosarcoma Tissues and Expressed in Osteosarcoma Cell Lines

**Figure 1:**
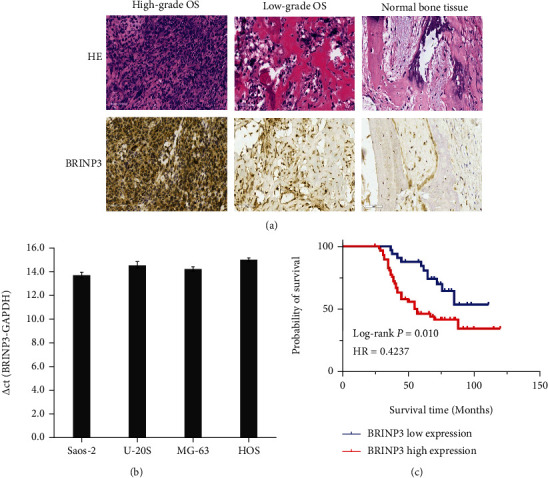
BRINP3 was upregulated in osteosarcoma tissues and osteosarcoma cell lines. (a) BRINP3 was overexpressed in the high-grade osteosarcoma tissue compared to the low-grade osteosarcoma and normal bone tissue by IHC (magnification, ×200). (b) Expression abundance of BRINP3 mRNA in different osteosarcoma cell lines by qRT-PCR. ΔC_T_ = target gene (BRINP3) C_T_ value − internal reference gene (GAPDH) C_T_ value. (c) Overall survival curves of low or high BRINP3 expression levels in patients with osteosarcoma.

To investigate the role of BRINP3 in osteosarcoma, immunohistochemical staining was performed on 92 independent osteosarcoma sections. Based on the analysis of the IHC results, a total of 59 (64.13%) patients showed high BRINP3 expression, while 33 (35.87%) patients displayed low BRINP3 expression. Furthermore, the correlation analysis showed that BRINP3 expression was associated with clinicopathological features such as the histological grade, tumor recurrence, and prognosis (Table 1) (Figures 1(a) and 1(c)). In addition, BRINP3 mRNA was expressed in human osteosarcoma cell lines (Figure 1(b)). These findings implied that BRINP3 was highly expressed in osteosarcoma tissues and expressed in osteosarcoma cell lines and was positively correlated with the prognosis of patients with osteosarcoma.

### 3.2. BRINP3 Knockdown Inhibits Osteosarcoma Cell Proliferation and Invasion

**Figure 2:**
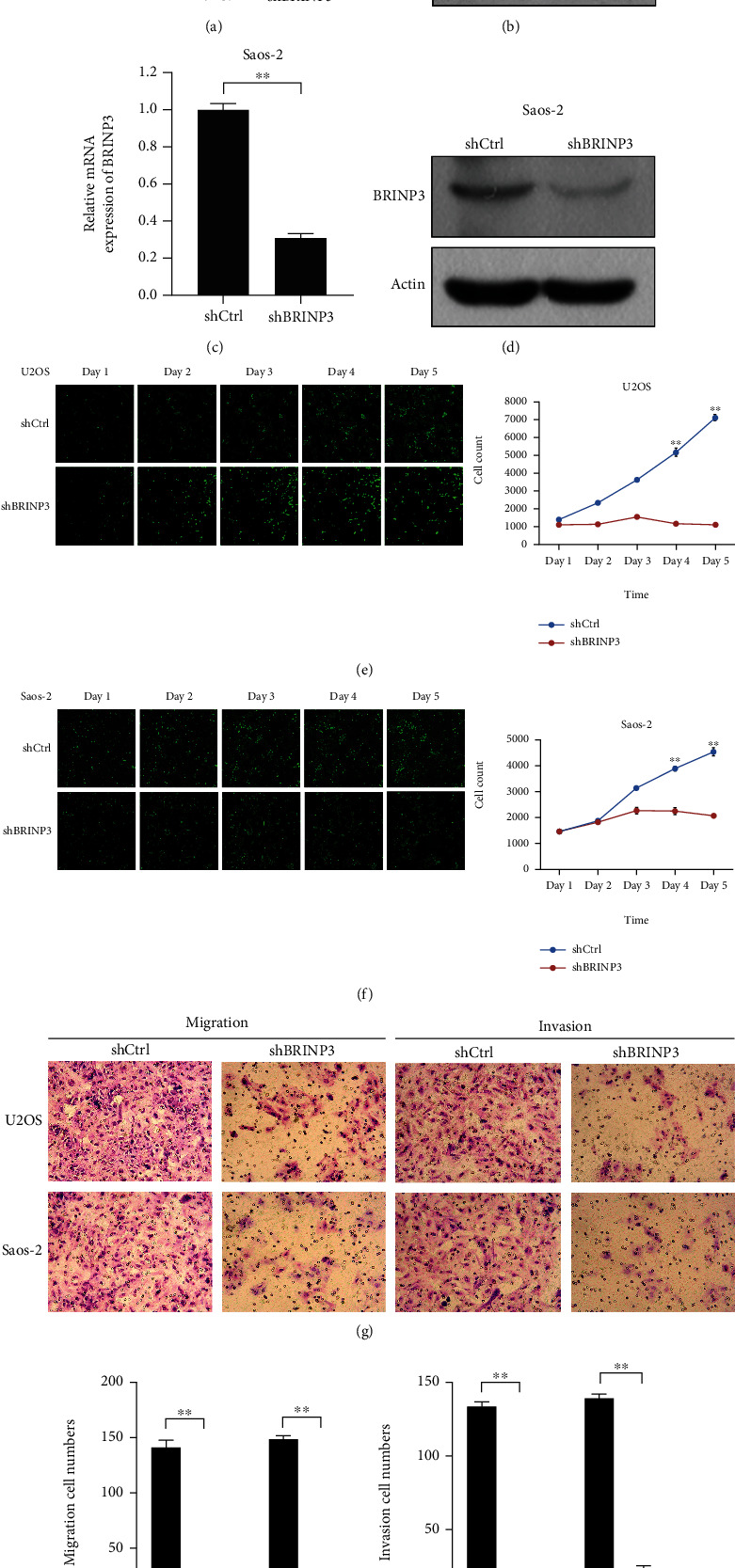
BRINP3 promotes the proliferative and metastatic capacity of osteosarcoma cell lines. (a, b) The mRNA level and protein level of BRINP3 after U2OS cells were transfected with shBRINP3, ^∗∗^*P* < 0.001. (c, d) The mRNA level and protein level of BRINP3 after Saos-2 cells were transfected with shBRINP3, ^∗∗^*P* < 0.001. (e, f) Cell proliferation was detected by Celigo image cytometry (magnification, ×100) after osteosarcoma cells were transfected with shBRINP3. (g) Images of the transwell assay (magnification, ×200) after osteosarcoma cells were transfected with shBRINP3. (h, i) Quantification of the transwell assay, ^∗∗^*P* < 0.001.

Considering the expression of BRINP3 in osteosarcoma cell lines, U2OS and Saos-2 cells were selected as the primary model for subsequent studies. We used a specific shRNA (shBRINP3) to silence the expression of BRINP3 and validated the shRNA knockdown efficiency. As shown in Figures 2(a) and 2(b) and Supplemental Figure S1A, the BRINP3 expression was significantly decreased in U2OS cells after transfection determined by qRT-PCR and western blotting. Likewise, shBRINP3 significantly decreased BRINP3 in Saos-2 cells (Figures 2(c) and 2(d) and Supplemental Figure S1A). Subsequently, the role that BRINP3 plays in the regulation of osteosarcoma cell proliferation was examined using the Celigo cell counting assay and MTT assay for 5 days. The Celigo and MTT assay data indicated that knockdown of BRINP3 suppressed the U2OS and Saos-2 cell proliferation rate on days 4 and 5 (Figure 2(e)), which attested to the inhibitory effect of BRINP3 knockdown on osteosarcoma cell proliferation. Moreover, the ability of BRINP3 to affect cell invasion was examined using transwell assay, which revealed that the invasive activity of U2OS and Saos-2 cells was significantly repressed following transfection with shBRINP3 compared with negative control cells (Figures 2(g)–2(i)). Together, these results suggested that BRINP3 promotes cell proliferation, migration, and invasion in osteosarcoma.

### 3.3. Identification of BRINP3-Interacting Proteins in Osteosarcoma Cells

**Figure 3:**
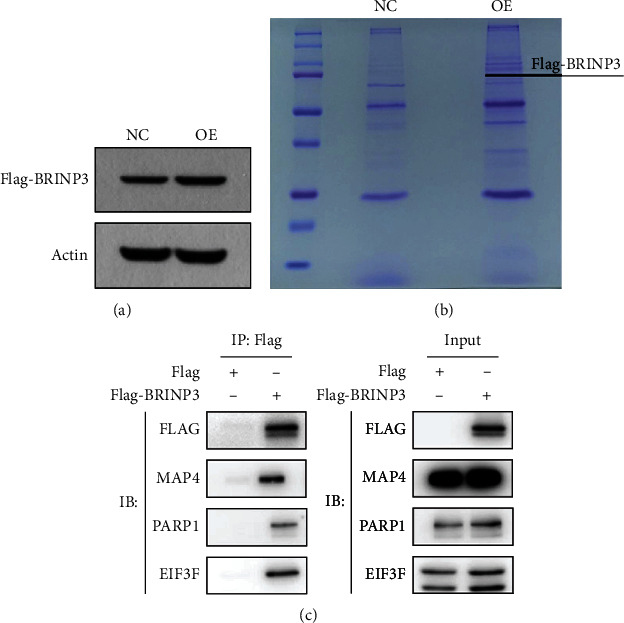
The interacting proteins with BRINP3. (a) The BRINP3 protein level of stable BRINP3-overexpressed cell lines by western blot. (b) Total cell lysates extracted from Flag-BRINP3 stably expressed cells were subjected to affinity purification by SDS-PAGE electrophoresis and Coomassie brilliant blue staining. (c) Co-IP of overexpressed Flag-BRINP3 and interacting proteins.

In order to investigate the underlying molecular mechanism of BRINP3 in regulating osteosarcoma progression, we first constructed a stable BRINP3-overexpressed U2OS cell line and performed immunoprecipitation assay, before using mass spectrometry to identify BRINP3-interacting proteins. As indicated in Figure 3(a) and Supplemental Figure S1B, BRINP3 was highly expressed in stably transfected U2OS cells. Affinity-purified total cell lysates extracted from stable expressing FLAG-BRINP3 cells were analyzed using mass spectrometry to determine the BRINP3-associated proteins (Figure 3(b)). Mass spectrometry identified a total of 262 BRINP3-interacting proteins based on the criteria of unique peptide ≥ 1 and peptide FDR < 0.01 (Table S3). The interaction between BRINP3 and 3 molecular markers including MAP4, EIF3F, and PARP1 was observed by immunoprecipitation in U2OS cells with overexpression of BRINP3 (Figure 3(c)).

### 3.4. BRINP3 Promotes Osteosarcoma Progression by Upregulating MAP4

**Figure 4:**
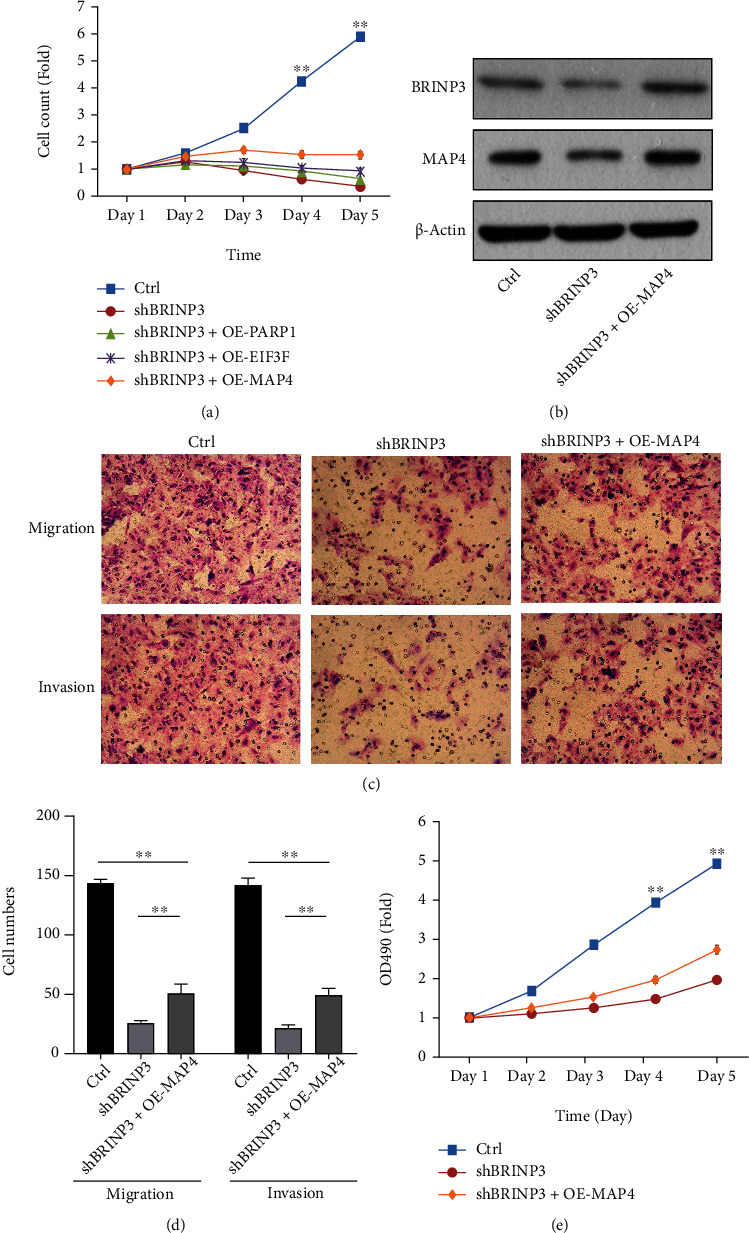
BRINP3 promotes osteosarcoma progression by upregulating MAP4 expression. (a) Celigo image cytometry assays demonstrated that MAP4 overexpression restored the greatest ability of proliferation in U2OS cells transfected with shBRINP3, ^∗∗^*P* < 0.001. (b) The protein level was detected by western blot assay in U2OS cells transfected with shBRINP3 and MAP4 overexpression. (c) Images of the transwell assay (magnification, ×200) after U2OS cells were transfected with shBRINP3 together with MAP4 overexpression. (d) Quantification of the transwell assay, ^∗∗^*P* < 0.001. (e) MTT assays showed that MAP4 overexpression restored the proliferation capability in U2OS cells transfected with shBRINP3, ^∗∗^*P* < 0.001.

In order to further clarify the regulatory relationship between BRINP3 and 3 molecular markers, we verified the proliferation ability of cells in different treatment groups by Celigo cell counting assay (Figure 4(a)). Interestingly, only MAP4 overexpression among the 3 molecular markers could reverse the inhibition of BRINP3 expression effects caused by shBRINP3. Western blotting results showed that BRINP3 downregulation in U2OS cells led to decreased expression of MAP4, suggesting that MAP4 was a downstream protein of BRINP3 (Figure 4(b) and Supplemental Figures S1C and S1D). Next, we investigated whether upregulated MAP4 was involved in BRINP3-induced osteosarcoma progression in vitro. Based on Figure 4(e), BRINP3 knockdown suppressed the proliferation ability of the U2OS cells significantly, whereas MAP4 overexpression could partially rescue the proliferation-inhibited effect caused by BRINP3 silencing. Moreover, BRINP3 silencing-mediated inhibition on invasiveness was also recovered by MAP4 overexpression (Figures 4(c) and 4(d)). Thus, our results demonstrated that BRINP3 promotes osteosarcoma progression possibly through the activation of MAP4.

## 4. Discussion

The formation and development of osteosarcoma are mainly attributed to the abnormal osteosarcoma cell proliferation and interference with differentiation [18]. It was demonstrated in a previous study that a lack of differentiation led to osteosarcoma progression and chemotherapy resistance [19]. Thus, in the present study, we evaluated bone differentiation-related gene BRINP3 expression in osteosarcoma and its role in the malignant progression of osteosarcoma. BRINP3 was highly expressed in 64.13% of human osteosarcoma tissues and negatively correlated with the prognosis of osteosarcoma. Interacting in vitro with MAP4 (microtubule-associated protein 4) at the protein level, BRINP3 might promote the proliferation and invasion of osteosarcoma cells by upregulating MAP4 expression. The results of this study show that BRINP3 not only expresses but also plays a critical role in osteosarcoma for the first time. Our research also suggested that BRINP3 might be a potential target for anti-osteosarcoma strategies.

BRINP3 was initially discovered as a gene activated by the bone morphogenetic protein (BMP) and retinoic acid (RA) signaling in the mouse brain. It is mainly and widely expressed in the nervous system, playing a role in the development of the nervous system [12]. In addition to neural tissues, fibroblasts, vascular smooth muscle cells (SMCs), cancer cells, and myoblastic cells also express this protein [11, 12, 16, 17]. Several studies have recently demonstrated a correlation between BRINP3 and human diseases including myocardial infarction, aggressive periodontitis, osteoblast differentiation, and tumor. Nevertheless, the association between BRINP3 and tumor is controversial. BRINP3 is considered a novel tumor suppressor gene in tongue squamous cell carcinoma [17] and might have similar biological functions in gastric cancer [20]. A deletion of the BRINP3 gene occurs in bladder cancer chromosome region candidate 1 (DBCCR1-like). The DBCCR1 gene has been identified as a candidate tumor suppressor in bladder cancer [21]. In contrast to these findings, overexpression of BRINP3 is observed in pituitary gonadotrope cells and BRINP3 promotes tumor cell proliferation, migration, and invasion, suggesting that it might be a critical factor in pituitary tumorigenesis [16]. Consistently, our immunohistochemistry results showed relatively strong BRINP3 staining in the osteosarcoma tissues and a positive correlation between BRINP3 and the clinical prognosis of osteosarcoma. Inhibition of BRINP3 could limit the proliferation and invasion of osteosarcoma cell lines, indicating the role of BRINP3 as an oncogene. However, through in vitro experiments, we also found that the level of BRINP3 mRNA was inconsistent with the aggressiveness of osteosarcoma cell lines (Figure 1(b)). Previous studies have shown that HOS was a highly aggressive osteosarcoma cell line with strong tumorigenicity, colony-forming ability, invasion, migration, and proliferation ability, compared with Saos-2, U2OS, and MG-63 [22]. In our research, what puzzles us is that BRINP3 mRNA seems to exist at the lowest level in HOS. There are many factors related to tumor progression, which comprise the acquired capabilities for sustaining proliferative signaling, evading growth suppressors, withstanding cell death, enabling replicative immortality, inducing/accessing vasculature, triggering invasion and metastasis, reestablishing cellular metabolism, and avoiding immune destruction [23]. Therefore, the reasons for the low level of BRINP3 mRNA in HOS need to be further studied in future research.

The mechanism of BRINP3 gene expression regulation remains unknown. Kuroiwa et al. [17] performed a whole-genome analysis of loss of heterozygosity (LOH) in tongue squamous cell carcinoma (SCC). In their study, LOH was observed in all of the 5 cases in the 1q31.1 region, yet only the BRINP3 gene was located in the 1q31.1 region. The results suggest that the BRINP3 gene may be a novel tumor suppressor gene specific to tongue SCC. Another study also showed that BRINP3 is an antioncogene in gastric cancer because of hypermethylation [20]. In vascular inflammation, inflammatory stimuli trigger the upregulated expression of BRINP3 through the NF-*κ*B and JNK signaling pathways, and BRINP3 increases the mRNAs of leukocyte adhesion molecules through the ROS–NF-*κ*B signaling pathway, thus enhancing monocyte adhesion to endothelial cells [24]. In the present study, BRINP3 interacts with MAP4 at the protein level. MAP4 could resist the inhibitory effect of downregulation of BRINP3 on the proliferation and invasion of osteosarcoma cells, suggesting that BRINP3 regulates the malignant progression of osteosarcoma cells at least partly through MAP4.

In human nonneuronal tissues, MAP4 is the primary nontubulin component of microtubule-associated protein [25, 26]. A pivotal role for MAP4 has been reported in microtubule stabilization and assembly [27]. So far, there have been few research on the association between MAP4 and human cancers. Researchers have found that phosphorylation of MAP4 could regulate microtubule stability and paclitaxel sensitivity, which could be used as a strategy to improve primary therapy for ovarian cancer [28, 29]. In terms of prostate cancer, MAP4 is also considered a potential biomarker for cancer detection and discrimination between prostate tumors with different malignancy and aggressiveness [30]. An elevated MAP4 expression in lung adenocarcinoma tissues was associated with differentiation, TNM stage, and pathological T stage [31]. Similarly, we found that BRINP3 could stimulate the proliferation and invasion of osteosarcoma by upregulating MAP4, suggesting that MAP4 might be related to the malignant progression of osteosarcoma. Additionally, MAP4 expression in mitochondria plays a crucial role in maintaining mitochondrial homeostasis [26]. It was also proven that the endogenous BRINP3 protein is located in the mitochondria of gonadotrope cells [16]. The study on the sublocalization of BRINP3 and MAP4 suggests the possibility of interaction between them. Heat-stable MAP4 has an asymmetric structure, characterized by an N-terminal projection (PJ) domain and a C-terminal microtubule-binding (MTB) domain. Research has revealed that the PJ domain regulates dynamic instability and that the MTB domain participates in cell cycle progression [31]. Whether BRINP3 regulates the phosphorylation of MAP4 needs to be further studied in future research.

In summary, our findings suggested that BRINP3 was overexpressed in human osteosarcoma and positively correlated with clinical prognosis. It promoted osteosarcoma proliferation and invasion through MAP4 in vitro. This supports that BRINP3 might play a role in human osteosarcoma tumorigenesis. Further studies are necessary to clarify the specific mechanism of BRINP3 regulating MAP4.

## 5. Conclusions

BRINP3 was highly expressed in human osteosarcoma tissues and negatively correlated with the prognosis of osteosarcoma. In vitro, downregulation of BRINP3 could inhibit the proliferation and invasion of osteosarcoma cell lines. BRINP3 downregulation might suppress the proliferation and invasion of osteosarcoma cells by inhibiting the expression of MAP4. Overall, our results revealed that BRINP3 functions as an oncogene in osteosarcoma through MAP4 and might be a potential biomarker for diagnosis and therapeutic targets for osteosarcoma.

## Figures and Tables

**Table 1:** Correlation between clinicopathological features and BRINP3 expression.

Characteristics	BRINP3 protein expression		*P* value
	Low expression *n*(%)	High expression *n*(%)	
Age			0.805
<20	25 (75.8%)	42 (71.2%)	
≥20	8 (24.2%)	17 (28.8%)	
Gender			0.028
Female	19 (57.6%)	20 (33.9%)	
Male	14 (42.4%)	39 (66.1%)	
Tumor size			0.13
<5	3 (9.1%)	1 (1.7%)	
≥5	30 (90.9%)	58 (98.3%)	
Tumor position			0.975
Femur	15 (45.5%)	30 (50.8%)	
Tibia	8 (24.2%)	13 (22.0%)	
Humerus	4 (12.1%)	7 (11.9%)	
Fibulars	2 (6.1%)	2 (3.4%)	
Irregular bone	4 (12.1%)	7 (11.9%)	
Histologic grade			<0.001
Low grade	24 (72.7%)	14 (23.7%)	
High grade	9 (27.3%)	45 (76.3%)	
Metastasis			0.081
Present	20 (60.6%)	23 (39.0%)	
Absent	9 (27.3%)	30 (50.8%)	
Unknown	4 (12.1%)	6 (10.2%)	
Recurrence			<0.001
Positive	4 (12.1%)	51 (86.4%)	
Negative	26 (78.8%)	5 (8.5%)	
Unknown	3 (9.1%)	3 (5.1%)	

## Data Availability

The authors certify that all the original data in this research could be obtained from a public database. All data generated or analyzed during this study are included in this article.

## Supplementary Materials

Supplementary Materials Supplementary Figure S1: quantitative western blot analysis of relative protein expression. (A) BRINP3 protein expression levels in the U2OS and Saos-2 cell lines transfected with shBRINP3 (quantification for Figures 2(b) and 2(d)). (B) BRINP3 protein expression levels of stable BRINP3-overexpressed cell lines (quantification for Figure 3(a)). (C) BRINP3 protein expression levels and (D) MAP4 protein expression levels in U2OS cells transfected with shBRINP3 and MAP4 overexpression (quantification for Figure 4(b)). Supplementary Table S1: the sequences of primers used in RT-PCR. Supplementary Table S2: antibody staining information for western blot. Supplementary Table S3: BRINP3-interacting proteins by mass spectrometry.
