# Supplementary material for: Overexpression of BRINP3 Predicts Poor Prognosis and Promotes Cancer Cell Proliferation and Migration via MAP4 in Osteosarcoma
Source: Dis Markers. 2022 Jul 7;2022:2698869. doi: 10.1155/2022/2698869 (PMC9282995; doi:10.1155/2022/2698869)
Supplement: Supplementary Materials — Supplementary Figure S1: quantitative western blot analysis of relative protein expression. (A) BRINP3 protein expression levels in the U2OS and Saos-2 cell lines transfected with shBRINP3 (quantification for Figures 2(b) and 2(d)). (B) BRINP3 protein expression levels of stable BRINP3-overexpressed cell lines (quantification for Figure 3(a)). (C) BRINP3 protein expression levels and (D) MAP4 protein expression levels in U2OS cells transfected with shBRINP3 and MAP4 overexpression (quantification for Figure 4(b)). Supplementary Table S1: the sequences of primers used in RT-PCR. Supplementary Table S2: antibody staining information for western blot. Supplementary Table S3: BRINP3-interacting proteins by mass spectrometry. [file 2698869.f1.zip › Table S2.docx]

| **Table S2. Antibody staining information for western blot** | | | | |
| --- | --- | --- | --- | --- |
| **Antibody against** | **Catalog number** | **Vendor** | **Dilution** | **Molecular Weight** |
| BRINP3 | 30374-1 | signalway antibody | 1:2000 | 70-100 kDa |
| GAPDH | sc-32233 | Santa-Cruz | 1:5000 | 146 kDa |
| β-actin | sc-69879 | Santa Cruz | 1:2000 | 43 kDa |
| MAP4 | 11229-1-AP | Proteintech | 1:600 | 210-240 kDa |
| EIF3F | ab176853 | abcam | 1:100 | 38 kDa |
| PARP1 | #9542 | CST | 1:1000 | 89116 kDa |
| Flag | F1804 | Sigma | 1:2000 | ~87 kDa |
