# Supplementary material for: Overexpression of BRINP3 Predicts Poor Prognosis and Promotes Cancer Cell Proliferation and Migration via MAP4 in Osteosarcoma
Source: Dis Markers. 2022 Jul 7;2022:2698869. doi: 10.1155/2022/2698869 (PMC9282995; doi:10.1155/2022/2698869)
Supplement: Supplementary Materials — Supplementary Figure S1: quantitative western blot analysis of relative protein expression. (A) BRINP3 protein expression levels in the U2OS and Saos-2 cell lines transfected with shBRINP3 (quantification for Figures 2(b) and 2(d)). (B) BRINP3 protein expression levels of stable BRINP3-overexpressed cell lines (quantification for Figure 3(a)). (C) BRINP3 protein expression levels and (D) MAP4 protein expression levels in U2OS cells transfected with shBRINP3 and MAP4 overexpression (quantification for Figure 4(b)). Supplementary Table S1: the sequences of primers used in RT-PCR. Supplementary Table S2: antibody staining information for western blot. Supplementary Table S3: BRINP3-interacting proteins by mass spectrometry. [file 2698869.f1.zip › Table S3.docx]

| **Table S3. BRINP3- interacting proteins by mass spectrometry （NC）** | | | | | | | | | | | |  | |  | |  | |  |  |
| --- | --- | --- | --- | --- | --- | --- | --- | --- | --- | --- | --- | --- | --- | --- | --- | --- | --- | --- | --- |
| Accession | | Gene Name | | Description | | | Coverage [%] | | | # Peptides | | # PSMs | | # Unique Peptides | | # AAs | | MW [kDa] | calc. pI |
| Q04917 | | YWHAH | | 14-3-3 protein eta OS=Homo sapiens OX=9606 GN=YWHAH PE=1 SV=4 | | | 6 | | | 1 | | 1 | | 1 | | 246 | | 28.2 | 4.84 |
| P61981 | | YWHAG | | 14-3-3 protein gamma OS=Homo sapiens OX=9606 GN=YWHAG PE=1 SV=2 | | | 6 | | | 1 | | 1 | | 1 | | 247 | | 28.3 | 4.89 |
| P27348 | | YWHAQ | | 14-3-3 protein theta OS=Homo sapiens OX=9606 GN=YWHAQ PE=1 SV=1 | | | 6 | | | 1 | | 1 | | 1 | | 245 | | 27.7 | 4.78 |
| P63104 | | YWHAZ | | 14-3-3 protein zeta/delta OS=Homo sapiens OX=9606 GN=YWHAZ PE=1 SV=1 | | | 6 | | | 1 | | 1 | | 1 | | 245 | | 27.7 | 4.79 |
| Q13200 | | PSMD2 | | 26S proteasome non-ATPase regulatory subunit 2 OS=Homo sapiens OX=9606 GN=PSMD2 PE=1 SV=3 | | | 6 | | | 3 | | 3 | | 3 | | 908 | | 100.1 | 5.2 |
| P62191 | | PSMC1 | | 26S proteasome regulatory subunit 4 OS=Homo sapiens OX=9606 GN=PSMC1 PE=1 SV=1 | | | 5 | | | 2 | | 2 | | 1 | | 440 | | 49.2 | 6.21 |
| P62195 | | PSMC5 | | 26S proteasome regulatory subunit 8 OS=Homo sapiens OX=9606 GN=PSMC5 PE=1 SV=1 | | | 6 | | | 2 | | 2 | | 1 | | 406 | | 45.6 | 7.55 |
| P62280 | | RPS11 | | 40S ribosomal protein S11 OS=Homo sapiens OX=9606 GN=RPS11 PE=1 SV=3 | | | 4 | | | 1 | | 1 | | 1 | | 158 | | 18.4 | 10.3 |
| P62277 | | RPS13 | | 40S ribosomal protein S13 OS=Homo sapiens OX=9606 GN=RPS13 PE=1 SV=2 | | | 5 | | | 1 | | 1 | | 1 | | 151 | | 17.2 | 10.54 |
| P62263 | | RPS14 | | 40S ribosomal protein S14 OS=Homo sapiens OX=9606 GN=RPS14 PE=1 SV=3 | | | 9 | | | 1 | | 1 | | 1 | | 151 | | 16.3 | 10.05 |
| P62269 | | RPS18 | | 40S ribosomal protein S18 OS=Homo sapiens OX=9606 GN=RPS18 PE=1 SV=3 | | | 15 | | | 2 | | 2 | | 2 | | 152 | | 17.7 | 10.99 |
| P23396 | | RPS3 | | 40S ribosomal protein S3 OS=Homo sapiens OX=9606 GN=RPS3 PE=1 SV=2 | | | 5 | | | 1 | | 1 | | 1 | | 243 | | 26.7 | 9.66 |
| P61247 | | RPS3A | | 40S ribosomal protein S3a OS=Homo sapiens OX=9606 GN=RPS3A PE=1 SV=2 | | | 4 | | | 1 | | 1 | | 1 | | 264 | | 29.9 | 9.73 |
| P62241 | | RPS8 | | 40S ribosomal protein S8 OS=Homo sapiens OX=9606 GN=RPS8 PE=1 SV=2 | | | 5 | | | 1 | | 1 | | 1 | | 208 | | 24.2 | 10.32 |
| P08865 | | RPSA | | 40S ribosomal protein SA OS=Homo sapiens OX=9606 GN=RPSA PE=1 SV=4 | | | 4 | | | 1 | | 1 | | 1 | | 295 | | 32.8 | 4.87 |
| P30050 | | RPL12 | | 60S ribosomal protein L12 OS=Homo sapiens OX=9606 GN=RPL12 PE=1 SV=1 | | | 15 | | | 2 | | 2 | | 2 | | 165 | | 17.8 | 9.42 |
| P26373 | | RPL13 | | 60S ribosomal protein L13 OS=Homo sapiens OX=9606 GN=RPL13 PE=1 SV=4 | | | 5 | | | 1 | | 1 | | 1 | | 211 | | 24.2 | 11.65 |
| P61313 | | RPL15 | | 60S ribosomal protein L15 OS=Homo sapiens OX=9606 GN=RPL15 PE=1 SV=2 | | | 4 | | | 1 | | 1 | | 1 | | 204 | | 24.1 | 11.62 |
| Q07020 | | RPL18 | | 60S ribosomal protein L18 OS=Homo sapiens OX=9606 GN=RPL18 PE=1 SV=2 | | | 11 | | | 2 | | 2 | | 2 | | 188 | | 21.6 | 11.72 |
| P61254 | | RPL26 | | 60S ribosomal protein L26 OS=Homo sapiens OX=9606 GN=RPL26 PE=1 SV=1 | | | 6 | | | 1 | | 1 | | 1 | | 145 | | 17.2 | 10.55 |
| P47914 | | RPL29 | | 60S ribosomal protein L29 OS=Homo sapiens OX=9606 GN=RPL29 PE=1 SV=2 | | | 9 | | | 1 | | 1 | | 1 | | 159 | | 17.7 | 11.66 |
| P62899 | | RPL31 | | 60S ribosomal protein L31 OS=Homo sapiens OX=9606 GN=RPL31 PE=1 SV=1 | | | 7 | | | 1 | | 1 | | 1 | | 125 | | 14.5 | 10.54 |
| P36578 | | RPL4 | | 60S ribosomal protein L4 OS=Homo sapiens OX=9606 GN=RPL4 PE=1 SV=5 | | | 3 | | | 1 | | 1 | | 1 | | 427 | | 47.7 | 11.06 |
| P18124 | | RPL7 | | 60S ribosomal protein L7 OS=Homo sapiens OX=9606 GN=RPL7 PE=1 SV=1 | | | 4 | | | 1 | | 1 | | 1 | | 248 | | 29.2 | 10.65 |
| P62424 | | RPL7A | | 60S ribosomal protein L7a OS=Homo sapiens OX=9606 GN=RPL7A PE=1 SV=2 | | | 7 | | | 2 | | 2 | | 2 | | 266 | | 30 | 10.61 |
| P60709 | | ACTB | | Actin, cytoplasmic 1 OS=Homo sapiens OX=9606 GN=ACTB PE=1 SV=1 | | | 27 | | | 9 | | 10 | | 9 | | 375 | | 41.7 | 5.48 |
| O14639 | | ABLIM1 | | Actin-binding LIM protein 1 OS=Homo sapiens OX=9606 GN=ABLIM1 PE=1 SV=3 | | | 2 | | | 1 | | 1 | | 1 | | 778 | | 87.6 | 8.59 |
| Q01518 | | CAP1 | | Adenylyl cyclase-associated protein 1 OS=Homo sapiens OX=9606 GN=CAP1 PE=1 SV=5 | | | 3 | | | 1 | | 1 | | 1 | | 475 | | 51.9 | 8.06 |
| P40123 | | CAP2 | | Adenylyl cyclase-associated protein 2 OS=Homo sapiens OX=9606 GN=CAP2 PE=1 SV=1 | | | 2 | | | 1 | | 1 | | 1 | | 477 | | 52.8 | 6.37 |
| P05141 | | SLC25A5 | | ADP/ATP translocase 2 OS=Homo sapiens OX=9606 GN=SLC25A5 PE=1 SV=7 | | | 3 | | | 1 | | 1 | | 1 | | 298 | | 32.8 | 9.69 |
| P18085 | | ARF4 | | ADP-ribosylation factor 4 OS=Homo sapiens OX=9606 GN=ARF4 PE=1 SV=3 | | | 4 | | | 1 | | 1 | | 1 | | 180 | | 20.5 | 7.14 |
| P06733 | | ENO1 | | Alpha-enolase OS=Homo sapiens OX=9606 GN=ENO1 PE=1 SV=2 | | | 4 | | | 2 | | 2 | | 2 | | 434 | | 47.1 | 7.39 |
| P07355 | | ANXA2 | | Annexin A2 OS=Homo sapiens OX=9606 GN=ANXA2 PE=1 SV=2 | | | 9 | | | 3 | | 3 | | 3 | | 339 | | 38.6 | 7.75 |
| Q9UKV3 | | ACIN1 | | Apoptotic chromatin condensation inducer in the nucleus OS=Homo sapiens OX=9606 GN=ACIN1 PE=1 SV=2 | | | 1 | | | 1 | | 1 | | 1 | | 1341 | | 151.8 | 6.43 |
| P25705 | | ATP5F1A | | ATP synthase subunit alpha, mitochondrial OS=Homo sapiens OX=9606 GN=ATP5F1A PE=1 SV=1 | | | 3 | | | 2 | | 2 | | 2 | | 553 | | 59.7 | 9.13 |
| P06576 | | ATP5F1B | | ATP synthase subunit beta, mitochondrial OS=Homo sapiens OX=9606 GN=ATP5F1B PE=1 SV=3 | | | 2 | | | 1 | | 1 | | 1 | | 529 | | 56.5 | 5.4 |
| Q08211 | | DHX9 | | ATP-dependent RNA helicase A OS=Homo sapiens OX=9606 GN=DHX9 PE=1 SV=4 | | | 1 | | | 1 | | 1 | | 1 | | 1270 | | 140.9 | 6.84 |
| Q9Y2J2 | | EPB41L3 | | Band 4.1-like protein 3 OS=Homo sapiens OX=9606 GN=EPB41L3 PE=1 SV=2 | | | 2 | | | 2 | | 2 | | 2 | | 1087 | | 120.6 | 5.19 |
| Q9NYF8 | | BCLAF1 | | Bcl-2-associated transcription factor 1 OS=Homo sapiens OX=9606 GN=BCLAF1 PE=1 SV=2 | | | 6 | | | 4 | | 5 | | 4 | | 920 | | 106.1 | 9.98 |
| P11586 | | MTHFD1 | | C-1-tetrahydrofolate synthase, cytoplasmic OS=Homo sapiens OX=9606 GN=MTHFD1 PE=1 SV=3 | | | 5 | | | 4 | | 4 | | 4 | | 935 | | 101.5 | 7.3 |
| P27708 | | CAD | | CAD protein OS=Homo sapiens OX=9606 GN=CAD PE=1 SV=3 | | | 1 | | | 1 | | 1 | | 1 | | 2225 | | 242.8 | 6.46 |
| P67870 | | CSNK2B | | Casein kinase II subunit beta OS=Homo sapiens OX=9606 GN=CSNK2B PE=1 SV=1 | | | 5 | | | 1 | | 1 | | 1 | | 215 | | 24.9 | 5.55 |
| Q03135 | | CAV1 | | Caveolin-1 OS=Homo sapiens OX=9606 GN=CAV1 PE=1 SV=4 | | | 14 | | | 2 | | 3 | | 2 | | 178 | | 20.5 | 6.02 |
| Q00610 | | CLTC | | Clathrin heavy chain 1 OS=Homo sapiens OX=9606 GN=CLTC PE=1 SV=5 | | | 2 | | | 4 | | 4 | | 4 | | 1675 | | 191.5 | 5.69 |
| P23528 | | CFL1 | | Cofilin-1 OS=Homo sapiens OX=9606 GN=CFL1 PE=1 SV=3 | | | 7 | | | 1 | | 1 | | 1 | | 166 | | 18.5 | 8.09 |
| Q14204 | | DYNC1H1 | | Cytoplasmic dynein 1 heavy chain 1 OS=Homo sapiens OX=9606 GN=DYNC1H1 PE=1 SV=5 | | | 0 | | | 2 | | 2 | | 2 | | 4646 | | 532.1 | 6.4 |
| Q07065 | | CKAP4 | | Cytoskeleton-associated protein 4 OS=Homo sapiens OX=9606 GN=CKAP4 PE=1 SV=2 | | | 1 | | | 1 | | 1 | | 1 | | 602 | | 66 | 5.92 |
| O43175 | | PHGDH | | D-3-phosphoglycerate dehydrogenase OS=Homo sapiens OX=9606 GN=PHGDH PE=1 SV=4 | | | 2 | | | 1 | | 1 | | 1 | | 533 | | 56.6 | 6.71 |
| Q16531 | | DDB1 | | DNA damage-binding protein 1 OS=Homo sapiens OX=9606 GN=DDB1 PE=1 SV=1 | | | 1 | | | 1 | | 1 | | 1 | | 1140 | | 126.9 | 5.26 |
| P78527 | | PRKDC | | DNA-dependent protein kinase catalytic subunit OS=Homo sapiens OX=9606 GN=PRKDC PE=1 SV=3 | | | 1 | | | 2 | | 2 | | 2 | | 4128 | | 468.8 | 7.12 |
| P04843 | | RPN1 | | Dolichyl-diphosphooligosaccharide--protein glycosyltransferase subunit 1 OS=Homo sapiens OX=9606 GN=RPN1 PE=1 SV=1 | | | 2 | | | 1 | | 1 | | 1 | | 607 | | 68.5 | 6.38 |
| Q16643 | | DBN1 | | Drebrin OS=Homo sapiens OX=9606 GN=DBN1 PE=1 SV=4 | | | 3 | | | 1 | | 1 | | 1 | | 649 | | 71.4 | 4.45 |
| Q14203 | | DCTN1 | | Dynactin subunit 1 OS=Homo sapiens OX=9606 GN=DCTN1 PE=1 SV=3 | | | 2 | | | 2 | | 2 | | 2 | | 1278 | | 141.6 | 5.81 |
| Q13561 | | DCTN2 | | Dynactin subunit 2 OS=Homo sapiens OX=9606 GN=DCTN2 PE=1 SV=4 | | | 2 | | | 1 | | 1 | | 1 | | 401 | | 44.2 | 5.21 |
| Q7Z6Z7 | | HUWE1 | | E3 ubiquitin-protein ligase HUWE1 OS=Homo sapiens OX=9606 GN=HUWE1 PE=1 SV=3 | | | 1 | | | 1 | | 1 | | 1 | | 4374 | | 481.6 | 5.22 |
| P19474 | | TRIM21 | | E3 ubiquitin-protein ligase TRIM21 OS=Homo sapiens OX=9606 GN=TRIM21 PE=1 SV=1 | | | 7 | | | 3 | | 3 | | 3 | | 475 | | 54.1 | 6.38 |
| Q05639 | | EEF1A2 | | Elongation factor 1-alpha 2 OS=Homo sapiens OX=9606 GN=EEF1A2 PE=1 SV=1 | | | 4 | | | 2 | | 2 | | 2 | | 463 | | 50.4 | 9.03 |
| P26641 | | EEF1G | | Elongation factor 1-gamma OS=Homo sapiens OX=9606 GN=EEF1G PE=1 SV=3 | | | 3 | | | 1 | | 1 | | 1 | | 437 | | 50.1 | 6.67 |
| P49411 | | TUFM | | Elongation factor Tu, mitochondrial OS=Homo sapiens OX=9606 GN=TUFM PE=1 SV=2 | | | 3 | | | 1 | | 1 | | 1 | | 452 | | 49.5 | 7.61 |
| P11021 | | HSPA5 | | Endoplasmic reticulum chaperone BiP OS=Homo sapiens OX=9606 GN=HSPA5 PE=1 SV=2 | | | 4 | | | 3 | | 3 | | 2 | | 654 | | 72.3 | 5.16 |
| P84090 | | ERH | | Enhancer of rudimentary homolog OS=Homo sapiens OX=9606 GN=ERH PE=1 SV=1 | | | 32 | | | 2 | | 2 | | 2 | | 104 | | 12.3 | 5.92 |
| P60842 | | EIF4A1 | | Eukaryotic initiation factor 4A-I OS=Homo sapiens OX=9606 GN=EIF4A1 PE=1 SV=1 | | | 10 | | | 3 | | 3 | | 3 | | 406 | | 46.1 | 5.48 |
| P38919 | | EIF4A3 | | Eukaryotic initiation factor 4A-III OS=Homo sapiens OX=9606 GN=EIF4A3 PE=1 SV=4 | | | 3 | | | 1 | | 1 | | 1 | | 411 | | 46.8 | 6.73 |
| O75821 | | EIF3G | | Eukaryotic translation initiation factor 3 subunit G OS=Homo sapiens OX=9606 GN=EIF3G PE=1 SV=2 | | | 3 | | | 1 | | 1 | | 1 | | 320 | | 35.6 | 6.13 |
| P23588 | | EIF4B | | Eukaryotic translation initiation factor 4B OS=Homo sapiens OX=9606 GN=EIF4B PE=1 SV=2 | | | 22 | | | 11 | | 22 | | 11 | | 611 | | 69.1 | 5.73 |
| Q16658 | | FSCN1 | | Fascin OS=Homo sapiens OX=9606 GN=FSCN1 PE=1 SV=3 | | | 3 | | | 1 | | 1 | | 1 | | 493 | | 54.5 | 7.24 |
| P49327 | | FASN | | Fatty acid synthase OS=Homo sapiens OX=9606 GN=FASN PE=1 SV=3 | | | 2 | | | 5 | | 5 | | 5 | | 2511 | | 273.3 | 6.44 |
| P21333 | | FLNA | | Filamin-A OS=Homo sapiens OX=9606 GN=FLNA PE=1 SV=4 | | | 15 | | | 28 | | 33 | | 26 | | 2647 | | 280.6 | 6.06 |
| O75369 | | FLNB | | Filamin-B OS=Homo sapiens OX=9606 GN=FLNB PE=1 SV=2 | | | 1 | | | 2 | | 2 | | 1 | | 2602 | | 278 | 5.73 |
| Q14315 | | FLNC | | Filamin-C OS=Homo sapiens OX=9606 GN=FLNC PE=1 SV=3 | | | 3 | | | 7 | | 8 | | 5 | | 2725 | | 290.8 | 5.97 |
| P51116 | | FXR2 | | Fragile X mental retardation syndrome-related protein 2 OS=Homo sapiens OX=9606 GN=FXR2 PE=1 SV=2 | | | 1 | | | 1 | | 1 | | 1 | | 673 | | 74.2 | 6.23 |
| P11413 | | G6PD | | Glucose-6-phosphate 1-dehydrogenase OS=Homo sapiens OX=9606 GN=G6PD PE=1 SV=4 | | | 2 | | | 1 | | 1 | | 1 | | 515 | | 59.2 | 6.84 |
| P47897 | | QARS | | Glutamine--tRNA ligase OS=Homo sapiens OX=9606 GN=QARS PE=1 SV=1 | | | 2 | | | 1 | | 1 | | 1 | | 775 | | 87.7 | 7.15 |
| P04406 | | GAPDH | | Glyceraldehyde-3-phosphate dehydrogenase OS=Homo sapiens OX=9606 GN=GAPDH PE=1 SV=3 | | | 9 | | | 3 | | 3 | | 3 | | 335 | | 36 | 8.46 |
| P62826 | | RAN | | GTP-binding nuclear protein Ran OS=Homo sapiens OX=9606 GN=RAN PE=1 SV=3 | | | 10 | | | 2 | | 2 | | 2 | | 216 | | 24.4 | 7.49 |
| Q5JWF2 | | GNAS | | Guanine nucleotide-binding protein G(s) subunit alpha isoforms XLas OS=Homo sapiens OX=9606 GN=GNAS PE=1 SV=2 | | | 1 | | | 1 | | 1 | | 1 | | 1037 | | 111 | 5.03 |
| P11142 | | HSPA8 | | Heat shock cognate 71 kDa protein OS=Homo sapiens OX=9606 GN=HSPA8 PE=1 SV=1 | | | 8 | | | 4 | | 4 | | 3 | | 646 | | 70.9 | 5.52 |
| P04792 | | HSPB1 | | Heat shock protein beta-1 OS=Homo sapiens OX=9606 GN=HSPB1 PE=1 SV=2 | | | 5 | | | 1 | | 1 | | 1 | | 205 | | 22.8 | 6.4 |
| P07900 | | HSP90AA1 | | Heat shock protein HSP 90-alpha OS=Homo sapiens OX=9606 GN=HSP90AA1 PE=1 SV=5 | | | 12 | | | 8 | | 8 | | 1 | | 732 | | 84.6 | 5.02 |
| P08238 | | HSP90AB1 | | Heat shock protein HSP 90-beta OS=Homo sapiens OX=9606 GN=HSP90AB1 PE=1 SV=4 | | | 20 | | | 13 | | 14 | | 6 | | 724 | | 83.2 | 5.03 |
| Q99729 | | HNRNPAB | | Heterogeneous nuclear ribonucleoprotein A/B OS=Homo sapiens OX=9606 GN=HNRNPAB PE=1 SV=2 | | | 4 | | | 1 | | 1 | | 1 | | 332 | | 36.2 | 8.21 |
| P09651 | | HNRNPA1 | | Heterogeneous nuclear ribonucleoprotein A1 OS=Homo sapiens OX=9606 GN=HNRNPA1 PE=1 SV=5 | | | 9 | | | 2 | | 3 | | 2 | | 372 | | 38.7 | 9.13 |
| Q14103 | | HNRNPD | | Heterogeneous nuclear ribonucleoprotein D0 OS=Homo sapiens OX=9606 GN=HNRNPD PE=1 SV=1 | | | 4 | | | 1 | | 1 | | 1 | | 355 | | 38.4 | 7.81 |
| P31943 | | HNRNPH1 | | Heterogeneous nuclear ribonucleoprotein H OS=Homo sapiens OX=9606 GN=HNRNPH1 PE=1 SV=4 | | | 9 | | | 3 | | 5 | | 3 | | 449 | | 49.2 | 6.3 |
| P61978 | | HNRNPK | | Heterogeneous nuclear ribonucleoprotein K OS=Homo sapiens OX=9606 GN=HNRNPK PE=1 SV=1 | | | 2 | | | 1 | | 1 | | 1 | | 463 | | 50.9 | 5.54 |
| Q00839 | | HNRNPU | | Heterogeneous nuclear ribonucleoprotein U OS=Homo sapiens OX=9606 GN=HNRNPU PE=1 SV=6 | | | 2 | | | 2 | | 2 | | 2 | | 825 | | 90.5 | 6 |
| P22626 | | HNRNPA2B1 | | Heterogeneous nuclear ribonucleoproteins A2/B1 OS=Homo sapiens OX=9606 GN=HNRNPA2B1 PE=1 SV=2 | | | 3 | | | 1 | | 1 | | 1 | | 353 | | 37.4 | 8.95 |
| P16403 | | HIST1H1C | | Histone H1.2 OS=Homo sapiens OX=9606 GN=HIST1H1C PE=1 SV=2 | | | 21 | | | 5 | | 5 | | 5 | | 213 | | 21.4 | 10.93 |
| P16401 | | HIST1H1B | | Histone H1.5 OS=Homo sapiens OX=9606 GN=HIST1H1B PE=1 SV=3 | | | 7 | | | 1 | | 1 | | 1 | | 226 | | 22.6 | 10.92 |
| Q96A08 | | HIST1H2BA | | Histone H2B type 1-A OS=Homo sapiens OX=9606 GN=HIST1H2BA PE=1 SV=3 | | | 14 | | | 2 | | 3 | | 2 | | 127 | | 14.2 | 10.32 |
| P68431 | | HIST1H3A | | Histone H3.1 OS=Homo sapiens OX=9606 GN=HIST1H3A PE=1 SV=2 | | | 5 | | | 1 | | 1 | | 1 | | 136 | | 15.4 | 11.12 |
| P62805 | | HIST1H4A | | Histone H4 OS=Homo sapiens OX=9606 GN=HIST1H4A PE=1 SV=2 | | | 21 | | | 2 | | 3 | | 2 | | 103 | | 11.4 | 11.36 |
| Q09028 | | RBBP4 | | Histone-binding protein RBBP4 OS=Homo sapiens OX=9606 GN=RBBP4 PE=1 SV=3 | | | 3 | | | 1 | | 1 | | 1 | | 425 | | 47.6 | 4.89 |
| P01859 | | IGHG2 | | Immunoglobulin heavy constant gamma 2 OS=Homo sapiens OX=9606 GN=IGHG2 PE=1 SV=2 | | | 3 | | | 1 | | 1 | | 1 | | 326 | | 35.9 | 7.59 |
| P01614 | | IGKV2D-40 | | Immunoglobulin kappa variable 2D-40 OS=Homo sapiens OX=9606 GN=IGKV2D-40 PE=1 SV=2 | | | 11 | | | 1 | | 2 | | 1 | | 121 | | 13.3 | 4.61 |
| P20839 | | IMPDH1 | | Inosine-5'-monophosphate dehydrogenase 1 OS=Homo sapiens OX=9606 GN=IMPDH1 PE=1 SV=2 | | | 2 | | | 1 | | 1 | | 1 | | 514 | | 55.4 | 6.9 |
| P12268 | | IMPDH2 | | Inosine-5'-monophosphate dehydrogenase 2 OS=Homo sapiens OX=9606 GN=IMPDH2 PE=1 SV=2 | | | 8 | | | 3 | | 3 | | 3 | | 514 | | 55.8 | 6.9 |
| P05556 | | ITGB1 | | Integrin beta-1 OS=Homo sapiens OX=9606 GN=ITGB1 PE=1 SV=2 | | | 1 | | | 1 | | 1 | | 1 | | 798 | | 88.4 | 5.39 |
| Q14766 | | LTBP1 | | Latent-transforming growth factor beta-binding protein 1 OS=Homo sapiens OX=9606 GN=LTBP1 PE=1 SV=4 | | | 1 | | | 1 | | 1 | | 1 | | 1721 | | 186.7 | 5.96 |
| P00338 | | LDHA | | L-lactate dehydrogenase A chain OS=Homo sapiens OX=9606 GN=LDHA PE=1 SV=2 | | | 3 | | | 1 | | 1 | | 1 | | 332 | | 36.7 | 8.27 |
| P07195 | | LDHB | | L-lactate dehydrogenase B chain OS=Homo sapiens OX=9606 GN=LDHB PE=1 SV=2 | | | 9 | | | 2 | | 3 | | 2 | | 334 | | 36.6 | 6.05 |
| Q9BQA1 | | WDR77 | | Methylosome protein 50 OS=Homo sapiens OX=9606 GN=WDR77 PE=1 SV=1 | | | 14 | | | 4 | | 4 | | 4 | | 342 | | 36.7 | 5.17 |
| P54105 | | CLNS1A | | Methylosome subunit pICln OS=Homo sapiens OX=9606 GN=CLNS1A PE=1 SV=1 | | | 5 | | | 1 | | 1 | | 1 | | 237 | | 26.2 | 4.11 |
| P46821 | | MAP1B | | Microtubule-associated protein 1B OS=Homo sapiens OX=9606 GN=MAP1B PE=1 SV=2 | | | 1 | | | 2 | | 2 | | 2 | | 2468 | | 270.5 | 4.81 |
| O43318 | | MAP3K7 | | Mitogen-activated protein kinase kinase kinase 7 OS=Homo sapiens OX=9606 GN=MAP3K7 PE=1 SV=1 | | | 3 | | | 1 | | 1 | | 1 | | 606 | | 67.2 | 7.11 |
| P60660 | | MYL6 | | Myosin light polypeptide 6 OS=Homo sapiens OX=9606 GN=MYL6 PE=1 SV=2 | | | 14 | | | 2 | | 2 | | 2 | | 151 | | 16.9 | 4.65 |
| O14950 | | MYL12B | | Myosin regulatory light chain 12B OS=Homo sapiens OX=9606 GN=MYL12B PE=1 SV=2 | | | 6 | | | 1 | | 1 | | 1 | | 172 | | 19.8 | 4.84 |
| P35579 | | MYH9 | | Myosin-9 OS=Homo sapiens OX=9606 GN=MYH9 PE=1 SV=4 | | | 9 | | | 17 | | 20 | | 17 | | 1960 | | 226.4 | 5.6 |
| Q14697 | | GANAB | | Neutral alpha-glucosidase AB OS=Homo sapiens OX=9606 GN=GANAB PE=1 SV=3 | | | 1 | | | 1 | | 1 | | 1 | | 944 | | 106.8 | 6.14 |
| P67809 | | YBX1 | | Nuclease-sensitive element-binding protein 1 OS=Homo sapiens OX=9606 GN=YBX1 PE=1 SV=3 | | | 9 | | | 1 | | 1 | | 1 | | 324 | | 35.9 | 9.88 |
| P19338 | | NCL | | Nucleolin OS=Homo sapiens OX=9606 GN=NCL PE=1 SV=3 | | | 9 | | | 7 | | 7 | | 7 | | 710 | | 76.6 | 4.7 |
| Q13162 | | PRDX4 | | Peroxiredoxin-4 OS=Homo sapiens OX=9606 GN=PRDX4 PE=1 SV=1 | | | 3 | | | 1 | | 1 | | 1 | | 271 | | 30.5 | 6.29 |
| P30041 | | PRDX6 | | Peroxiredoxin-6 OS=Homo sapiens OX=9606 GN=PRDX6 PE=1 SV=3 | | | 6 | | | 1 | | 1 | | 1 | | 224 | | 25 | 6.38 |
| Q9NSD9 | | FARSB | | Phenylalanine--tRNA ligase beta subunit OS=Homo sapiens OX=9606 GN=FARSB PE=1 SV=3 | | | 2 | | | 1 | | 1 | | 1 | | 589 | | 66.1 | 6.84 |
| Q00325 | | SLC25A3 | | Phosphate carrier protein, mitochondrial OS=Homo sapiens OX=9606 GN=SLC25A3 PE=1 SV=2 | | | 3 | | | 1 | | 1 | | 1 | | 362 | | 40.1 | 9.38 |
| Q15149 | | PLEC | | Plectin OS=Homo sapiens OX=9606 GN=PLEC PE=1 SV=3 | | | 7 | | | 29 | | 32 | | 29 | | 4684 | | 531.5 | 5.96 |
| P57721 | | PCBP3 | | Poly(rC)-binding protein 3 OS=Homo sapiens OX=9606 GN=PCBP3 PE=2 SV=2 | | | 3 | | | 1 | | 1 | | 1 | | 371 | | 39.4 | 8.07 |
| O94906 | | PRPF6 | | Pre-mRNA-processing factor 6 OS=Homo sapiens OX=9606 GN=PRPF6 PE=1 SV=1 | | | 1 | | | 1 | | 1 | | 1 | | 941 | | 106.9 | 8.25 |
| Q8WUM4 | | PDCD6IP | | Programmed cell death 6-interacting protein OS=Homo sapiens OX=9606 GN=PDCD6IP PE=1 SV=1 | | | 1 | | | 1 | | 1 | | 1 | | 868 | | 96 | 6.52 |
| Q99623 | | PHB2 | | Prohibitin-2 OS=Homo sapiens OX=9606 GN=PHB2 PE=1 SV=2 | | | 3 | | | 1 | | 1 | | 1 | | 299 | | 33.3 | 9.83 |
| Q99873 | | PRMT1 | | Protein arginine N-methyltransferase 1 OS=Homo sapiens OX=9606 GN=PRMT1 PE=1 SV=3 | | | 3 | | | 1 | | 1 | | 1 | | 371 | | 42.4 | 5.35 |
| O14744 | | PRMT5 | | Protein arginine N-methyltransferase 5 OS=Homo sapiens OX=9606 GN=PRMT5 PE=1 SV=4 | | | 13 | | | 7 | | 9 | | 7 | | 637 | | 72.6 | 6.29 |
| O75688 | | PPM1B | | Protein phosphatase 1B OS=Homo sapiens OX=9606 GN=PPM1B PE=1 SV=1 | | | 12 | | | 5 | | 5 | | 5 | | 479 | | 52.6 | 5.05 |
| Q9HCE1 | | MOV10 | | Putative helicase MOV-10 OS=Homo sapiens OX=9606 GN=MOV10 PE=1 SV=2 | | | 1 | | | 1 | | 1 | | 1 | | 1003 | | 113.6 | 8.82 |
| P30613 | | PKLR | | Pyruvate kinase PKLR OS=Homo sapiens OX=9606 GN=PKLR PE=1 SV=2 | | | 2 | | | 1 | | 1 | | 1 | | 574 | | 61.8 | 7.74 |
| P35250 | | RFC2 | | Replication factor C subunit 2 OS=Homo sapiens OX=9606 GN=RFC2 PE=1 SV=3 | | | 4 | | | 1 | | 1 | | 1 | | 354 | | 39.1 | 6.44 |
| P98175 | | RBM10 | | RNA-binding protein 10 OS=Homo sapiens OX=9606 GN=RBM10 PE=1 SV=3 | | | 3 | | | 3 | | 4 | | 3 | | 930 | | 103.5 | 5.97 |
| Q9NWH9 | | SLTM | | SAFB-like transcription modulator OS=Homo sapiens OX=9606 GN=SLTM PE=1 SV=2 | | | 2 | | | 1 | | 1 | | 1 | | 1034 | | 117.1 | 7.87 |
| Q6P3W7 | | SCYL2 | | SCY1-like protein 2 OS=Homo sapiens OX=9606 GN=SCYL2 PE=1 SV=1 | | | 4 | | | 3 | | 3 | | 3 | | 929 | | 103.6 | 8.22 |
| Q13247 | | SRSF6 | | Serine/arginine-rich splicing factor 6 OS=Homo sapiens OX=9606 GN=SRSF6 PE=1 SV=2 | | | 5 | | | 2 | | 2 | | 2 | | 344 | | 39.6 | 11.43 |
| Q16629 | | SRSF7 | | Serine/arginine-rich splicing factor 7 OS=Homo sapiens OX=9606 GN=SRSF7 PE=1 SV=1 | | | 9 | | | 2 | | 2 | | 2 | | 238 | | 27.4 | 11.82 |
| Q15208 | | STK38 | | Serine/threonine-protein kinase 38 OS=Homo sapiens OX=9606 GN=STK38 PE=1 SV=1 | | | 3 | | | 1 | | 1 | | 1 | | 465 | | 54.2 | 7.15 |
| P50454 | | SERPINH1 | | Serpin H1 OS=Homo sapiens OX=9606 GN=SERPINH1 PE=1 SV=2 | | | 5 | | | 2 | | 2 | | 2 | | 418 | | 46.4 | 8.69 |
| P02768 | | ALB | | Serum albumin OS=Homo sapiens OX=9606 GN=ALB PE=1 SV=2 | | | 2 | | | 1 | | 1 | | 1 | | 609 | | 69.3 | 6.28 |
| Q9Y5M8 | | SRPRB | | Signal recognition particle receptor subunit beta OS=Homo sapiens OX=9606 GN=SRPRB PE=1 SV=3 | | | 7 | | | 1 | | 1 | | 1 | | 271 | | 29.7 | 9.04 |
| P62316 | | SNRPD2 | | Small nuclear ribonucleoprotein Sm D2 OS=Homo sapiens OX=9606 GN=SNRPD2 PE=1 SV=1 | | | 8 | | | 1 | | 1 | | 1 | | 118 | | 13.5 | 9.91 |
| Q13813 | | SPTAN1 | | Spectrin alpha chain, non-erythrocytic 1 OS=Homo sapiens OX=9606 GN=SPTAN1 PE=1 SV=3 | | | 11 | | | 24 | | 26 | | 24 | | 2472 | | 284.4 | 5.35 |
| Q01082 | | SPTBN1 | | Spectrin beta chain, non-erythrocytic 1 OS=Homo sapiens OX=9606 GN=SPTBN1 PE=1 SV=2 | | | 10 | | | 23 | | 25 | | 23 | | 2364 | | 274.4 | 5.57 |
| Q9BUA3 | | C11orf84 | | SPIN1-docking protein OS=Homo sapiens OX=9606 GN=C11orf84 PE=1 SV=3 | | | 2 | | | 1 | | 1 | | 1 | | 381 | | 41 | 5.01 |
| Q9Y657 | | SPIN1 | | Spindlin-1 OS=Homo sapiens OX=9606 GN=SPIN1 PE=1 SV=3 | | | 4 | | | 1 | | 1 | | 1 | | 262 | | 29.6 | 6.96 |
| Q5JUX0 | | SPIN3 | | Spindlin-3 OS=Homo sapiens OX=9606 GN=SPIN3 PE=1 SV=1 | | | 4 | | | 1 | | 1 | | 1 | | 258 | | 29.2 | 6.8 |
| P23246 | | SFPQ | | Splicing factor, proline- and glutamine-rich OS=Homo sapiens OX=9606 GN=SFPQ PE=1 SV=2 | | | 1 | | | 1 | | 1 | | 1 | | 707 | | 76.1 | 9.44 |
| P38646 | | HSPA9 | | Stress-70 protein, mitochondrial OS=Homo sapiens OX=9606 GN=HSPA9 PE=1 SV=2 | | | 3 | | | 2 | | 2 | | 2 | | 679 | | 73.6 | 6.16 |
| P48643 | | CCT5 | | T-complex protein 1 subunit epsilon OS=Homo sapiens OX=9606 GN=CCT5 PE=1 SV=1 | | | 2 | | | 1 | | 1 | | 1 | | 541 | | 59.6 | 5.66 |
| Q99832 | | CCT7 | | T-complex protein 1 subunit eta OS=Homo sapiens OX=9606 GN=CCT7 PE=1 SV=2 | | | 2 | | | 1 | | 1 | | 1 | | 543 | | 59.3 | 7.65 |
| Q15750 | | TAB1 | | TGF-beta-activated kinase 1 and MAP3K7-binding protein 1 OS=Homo sapiens OX=9606 GN=TAB1 PE=1 SV=1 | | | 2 | | | 1 | | 1 | | 1 | | 504 | | 54.6 | 5.52 |
| Q9Y2W1 | | THRAP3 | | Thyroid hormone receptor-associated protein 3 OS=Homo sapiens OX=9606 GN=THRAP3 PE=1 SV=2 | | | 9 | | | 8 | | 8 | | 8 | | 955 | | 108.6 | 10.15 |
| P02786 | | TFRC | | Transferrin receptor protein 1 OS=Homo sapiens OX=9606 GN=TFRC PE=1 SV=2 | | | 1 | | | 1 | | 1 | | 1 | | 760 | | 84.8 | 6.61 |
| Q13595 | | TRA2A | | Transformer-2 protein homolog alpha OS=Homo sapiens OX=9606 GN=TRA2A PE=1 SV=1 | | | 4 | | | 1 | | 1 | | 1 | | 282 | | 32.7 | 11.27 |
| P62995 | | TRA2B | | Transformer-2 protein homolog beta OS=Homo sapiens OX=9606 GN=TRA2B PE=1 SV=1 | | | 7 | | | 2 | | 2 | | 2 | | 288 | | 33.6 | 11.25 |
| P55072 | | VCP | | Transitional endoplasmic reticulum ATPase OS=Homo sapiens OX=9606 GN=VCP PE=1 SV=4 | | | 2 | | | 1 | | 1 | | 1 | | 806 | | 89.3 | 5.26 |
| P40939 | | HADHA | | Trifunctional enzyme subunit alpha, mitochondrial OS=Homo sapiens OX=9606 GN=HADHA PE=1 SV=2 | | | 5 | | | 3 | | 3 | | 3 | | 763 | | 82.9 | 9.04 |
| P55084 | | HADHB | | Trifunctional enzyme subunit beta, mitochondrial OS=Homo sapiens OX=9606 GN=HADHB PE=1 SV=3 | | | 2 | | | 1 | | 1 | | 1 | | 474 | | 51.3 | 9.41 |
| P0CI25 | | TRIM49 | | Tripartite motif-containing protein 49 OS=Homo sapiens OX=9606 GN=TRIM49 PE=2 SV=1 | | | 5 | | | 1 | | 1 | | 1 | | 452 | | 52.9 | 7.58 |
| P35030 | | PRSS3 | | Trypsin-3 OS=Homo sapiens OX=9606 GN=PRSS3 PE=1 SV=2 | | | 4 | | | 1 | | 1 | | 1 | | 304 | | 32.5 | 7.49 |
| P68363 | | TUBA1B | | Tubulin alpha-1B chain OS=Homo sapiens OX=9606 GN=TUBA1B PE=1 SV=1 | | | 11 | | | 5 | | 5 | | 5 | | 451 | | 50.1 | 5.06 |
| P07437 | | TUBB | | Tubulin beta chain OS=Homo sapiens OX=9606 GN=TUBB PE=1 SV=2 | | | 16 | | | 6 | | 8 | | 1 | | 444 | | 49.6 | 4.89 |
| P68371 | | TUBB4B | | Tubulin beta-4B chain OS=Homo sapiens OX=9606 GN=TUBB4B PE=1 SV=1 | | | 16 | | | 6 | | 8 | | 1 | | 445 | | 49.8 | 4.89 |
| Q9BUF5 | | TUBB6 | | Tubulin beta-6 chain OS=Homo sapiens OX=9606 GN=TUBB6 PE=1 SV=1 | | | 6 | | | 2 | | 3 | | 1 | | 446 | | 49.8 | 4.88 |
| O75643 | | SNRNP200 | | U5 small nuclear ribonucleoprotein 200 kDa helicase OS=Homo sapiens OX=9606 GN=SNRNP200 PE=1 SV=2 | | | 0 | | | 1 | | 1 | | 1 | | 2136 | | 244.4 | 6.06 |
| Q9Y4E8 | | USP15 | | Ubiquitin carboxyl-terminal hydrolase 15 OS=Homo sapiens OX=9606 GN=USP15 PE=1 SV=3 | | | 1 | | | 1 | | 1 | | 1 | | 981 | | 112.3 | 5.22 |
| O15294 | | OGT | | UDP-N-acetylglucosamine--peptide N-acetylglucosaminyltransferase 110 kDa subunit OS=Homo sapiens OX=9606 GN=OGT PE=1 SV=3 | | | 2 | | | 1 | | 1 | | 1 | | 1046 | | 116.9 | 6.7 |
| O00159 | | MYO1C | | Unconventional myosin-Ic OS=Homo sapiens OX=9606 GN=MYO1C PE=1 SV=4 | | | 1 | | | 1 | | 1 | | 1 | | 1063 | | 121.6 | 9.41 |
| Q9ULV0 | | MYO5B | | Unconventional myosin-Vb OS=Homo sapiens OX=9606 GN=MYO5B PE=1 SV=3 | | | 2 | | | 3 | | 3 | | 3 | | 1848 | | 213.5 | 7.2 |
| P08670 | | VIM | | Vimentin OS=Homo sapiens OX=9606 GN=VIM PE=1 SV=4 | | | 4 | | | 2 | | 2 | | 1 | | 466 | | 53.6 | 5.12 |
| P16989 | | YBX3 | | Y-box-binding protein 3 OS=Homo sapiens OX=9606 GN=YBX3 PE=1 SV=4 | | | 5 | | | 1 | | 2 | | 1 | | 372 | | 40.1 | 9.77 |
| Q96N22 | | ZNF681 | | Zinc finger protein 681 OS=Homo sapiens OX=9606 GN=ZNF681 PE=2 SV=2 | | | 4 | | | 1 | | 1 | | 1 | | 645 | | 75 | 9.09 |
| **Table S3. BRINP3- interacting proteins by mass spectrometry (OE)** | | | | | | | |  |  | |  | |  | |  | |  |  |  |
| Accession | Gene Name | | Description | | Coverage [%] | # Peptides | | # PSMs | # Unique Peptides | | # AAs | | MW [kDa] | | calc. pI | |  |  |  |
| P31946 | YWHAB | | 14-3-3 protein beta/alpha OS=Homo sapiens OX=9606 GN=YWHAB PE=1 SV=3 | | 17 | 4 | | 5 | 2 | | 246 | | 28.1 | | 4.83 | |  |  |  |
| P62258 | YWHAE | | 14-3-3 protein epsilon OS=Homo sapiens OX=9606 GN=YWHAE PE=1 SV=1 | | 12 | 3 | | 3 | 2 | | 255 | | 29.2 | | 4.74 | |  |  |  |
| Q04917 | YWHAH | | 14-3-3 protein eta OS=Homo sapiens OX=9606 GN=YWHAH PE=1 SV=4 | | 16 | 4 | | 4 | 2 | | 246 | | 28.2 | | 4.84 | |  |  |  |
| P61981 | YWHAG | | 14-3-3 protein gamma OS=Homo sapiens OX=9606 GN=YWHAG PE=1 SV=2 | | 11 | 3 | | 3 | 1 | | 247 | | 28.3 | | 4.89 | |  |  |  |
| P31947 | SFN | | 14-3-3 protein sigma OS=Homo sapiens OX=9606 GN=SFN PE=1 SV=1 | | 12 | 3 | | 3 | 1 | | 248 | | 27.8 | | 4.74 | |  |  |  |
| P27348 | YWHAQ | | 14-3-3 protein theta OS=Homo sapiens OX=9606 GN=YWHAQ PE=1 SV=1 | | 12 | 3 | | 3 | 1 | | 245 | | 27.7 | | 4.78 | |  |  |  |
| P63104 | YWHAZ | | 14-3-3 protein zeta/delta OS=Homo sapiens OX=9606 GN=YWHAZ PE=1 SV=1 | | 13 | 3 | | 3 | 2 | | 245 | | 27.7 | | 4.79 | |  |  |  |
| Q13200 | PSMD2 | | 26S proteasome non-ATPase regulatory subunit 2 OS=Homo sapiens OX=9606 GN=PSMD2 PE=1 SV=3 | | 6 | 3 | | 3 | 3 | | 908 | | 100.1 | | 5.2 | |  |  |  |
| P62191 | PSMC1 | | 26S proteasome regulatory subunit 4 OS=Homo sapiens OX=9606 GN=PSMC1 PE=1 SV=1 | | 3 | 1 | | 1 | 1 | | 440 | | 49.2 | | 6.21 | |  |  |  |
| P35998 | PSMC2 | | 26S proteasome regulatory subunit 7 OS=Homo sapiens OX=9606 GN=PSMC2 PE=1 SV=3 | | 3 | 1 | | 1 | 1 | | 433 | | 48.6 | | 5.95 | |  |  |  |
| P62280 | RPS11 | | 40S ribosomal protein S11 OS=Homo sapiens OX=9606 GN=RPS11 PE=1 SV=3 | | 4 | 1 | | 1 | 1 | | 158 | | 18.4 | | 10.3 | |  |  |  |
| P62277 | RPS13 | | 40S ribosomal protein S13 OS=Homo sapiens OX=9606 GN=RPS13 PE=1 SV=2 | | 5 | 1 | | 1 | 1 | | 151 | | 17.2 | | 10.54 | |  |  |  |
| P62263 | RPS14 | | 40S ribosomal protein S14 OS=Homo sapiens OX=9606 GN=RPS14 PE=1 SV=3 | | 7 | 1 | | 1 | 1 | | 151 | | 16.3 | | 10.05 | |  |  |  |
| P62244 | RPS15A | | 40S ribosomal protein S15a OS=Homo sapiens OX=9606 GN=RPS15A PE=1 SV=2 | | 7 | 1 | | 1 | 1 | | 130 | | 14.8 | | 10.13 | |  |  |  |
| P62269 | RPS18 | | 40S ribosomal protein S18 OS=Homo sapiens OX=9606 GN=RPS18 PE=1 SV=3 | | 7 | 1 | | 1 | 1 | | 152 | | 17.7 | | 10.99 | |  |  |  |
| P39019 | RPS19 | | 40S ribosomal protein S19 OS=Homo sapiens OX=9606 GN=RPS19 PE=1 SV=2 | | 6 | 1 | | 1 | 1 | | 145 | | 16.1 | | 10.32 | |  |  |  |
| P15880 | RPS2 | | 40S ribosomal protein S2 OS=Homo sapiens OX=9606 GN=RPS2 PE=1 SV=2 | | 3 | 1 | | 1 | 1 | | 293 | | 31.3 | | 10.24 | |  |  |  |
| P62851 | RPS25 | | 40S ribosomal protein S25 OS=Homo sapiens OX=9606 GN=RPS25 PE=1 SV=1 | | 14 | 2 | | 2 | 2 | | 125 | | 13.7 | | 10.11 | |  |  |  |
| P61247 | RPS3A | | 40S ribosomal protein S3a OS=Homo sapiens OX=9606 GN=RPS3A PE=1 SV=2 | | 4 | 1 | | 1 | 1 | | 264 | | 29.9 | | 9.73 | |  |  |  |
| P62701 | RPS4X | | 40S ribosomal protein S4, X isoform OS=Homo sapiens OX=9606 GN=RPS4X PE=1 SV=2 | | 3 | 1 | | 1 | 1 | | 263 | | 29.6 | | 10.15 | |  |  |  |
| P46781 | RPS9 | | 40S ribosomal protein S9 OS=Homo sapiens OX=9606 GN=RPS9 PE=1 SV=3 | | 4 | 1 | | 1 | 1 | | 194 | | 22.6 | | 10.65 | |  |  |  |
| P08865 | RPSA | | 40S ribosomal protein SA OS=Homo sapiens OX=9606 GN=RPSA PE=1 SV=4 | | 4 | 1 | | 1 | 1 | | 295 | | 32.8 | | 4.87 | |  |  |  |
| P10809 | HSPD1 | | 60 kDa heat shock protein, mitochondrial OS=Homo sapiens OX=9606 GN=HSPD1 PE=1 SV=2 | | 2 | 1 | | 1 | 1 | | 573 | | 61 | | 5.87 | |  |  |  |
| P05388 | RPLP0 | | 60S acidic ribosomal protein P0 OS=Homo sapiens OX=9606 GN=RPLP0 PE=1 SV=1 | | 2 | 1 | | 1 | 1 | | 317 | | 34.3 | | 5.97 | |  |  |  |
| P62906 | RPL10A | | 60S ribosomal protein L10a OS=Homo sapiens OX=9606 GN=RPL10A PE=1 SV=2 | | 7 | 2 | | 2 | 2 | | 217 | | 24.8 | | 9.94 | |  |  |  |
| P30050 | RPL12 | | 60S ribosomal protein L12 OS=Homo sapiens OX=9606 GN=RPL12 PE=1 SV=1 | | 5 | 1 | | 1 | 1 | | 165 | | 17.8 | | 9.42 | |  |  |  |
| P26373 | RPL13 | | 60S ribosomal protein L13 OS=Homo sapiens OX=9606 GN=RPL13 PE=1 SV=4 | | 5 | 1 | | 1 | 1 | | 211 | | 24.2 | | 11.65 | |  |  |  |
| P40429 | RPL13A | | 60S ribosomal protein L13a OS=Homo sapiens OX=9606 GN=RPL13A PE=1 SV=2 | | 4 | 1 | | 1 | 1 | | 203 | | 23.6 | | 10.93 | |  |  |  |
| P50914 | RPL14 | | 60S ribosomal protein L14 OS=Homo sapiens OX=9606 GN=RPL14 PE=1 SV=4 | | 5 | 1 | | 1 | 1 | | 215 | | 23.4 | | 10.93 | |  |  |  |
| P61313 | RPL15 | | 60S ribosomal protein L15 OS=Homo sapiens OX=9606 GN=RPL15 PE=1 SV=2 | | 4 | 1 | | 1 | 1 | | 204 | | 24.1 | | 11.62 | |  |  |  |
| Q07020 | RPL18 | | 60S ribosomal protein L18 OS=Homo sapiens OX=9606 GN=RPL18 PE=1 SV=2 | | 11 | 2 | | 2 | 2 | | 188 | | 21.6 | | 11.72 | |  |  |  |
| P84098 | RPL19 | | 60S ribosomal protein L19 OS=Homo sapiens OX=9606 GN=RPL19 PE=1 SV=1 | | 5 | 1 | | 1 | 1 | | 196 | | 23.5 | | 11.47 | |  |  |  |
| P62750 | RPL23A | | 60S ribosomal protein L23a OS=Homo sapiens OX=9606 GN=RPL23A PE=1 SV=1 | | 8 | 1 | | 1 | 1 | | 156 | | 17.7 | | 10.45 | |  |  |  |
| P39023 | RPL3 | | 60S ribosomal protein L3 OS=Homo sapiens OX=9606 GN=RPL3 PE=1 SV=2 | | 2 | 1 | | 1 | 1 | | 403 | | 46.1 | | 10.18 | |  |  |  |
| P36578 | RPL4 | | 60S ribosomal protein L4 OS=Homo sapiens OX=9606 GN=RPL4 PE=1 SV=5 | | 4 | 2 | | 2 | 2 | | 427 | | 47.7 | | 11.06 | |  |  |  |
| P46777 | RPL5 | | 60S ribosomal protein L5 OS=Homo sapiens OX=9606 GN=RPL5 PE=1 SV=3 | | 3 | 1 | | 1 | 1 | | 297 | | 34.3 | | 9.72 | |  |  |  |
| Q02878 | RPL6 | | 60S ribosomal protein L6 OS=Homo sapiens OX=9606 GN=RPL6 PE=1 SV=3 | | 2 | 1 | | 1 | 1 | | 288 | | 32.7 | | 10.58 | |  |  |  |
| P62917 | RPL8 | | 60S ribosomal protein L8 OS=Homo sapiens OX=9606 GN=RPL8 PE=1 SV=2 | | 4 | 1 | | 1 | 1 | | 257 | | 28 | | 11.03 | |  |  |  |
| P52209 | PGD | | 6-phosphogluconate dehydrogenase, decarboxylating OS=Homo sapiens OX=9606 GN=PGD PE=1 SV=3 | | 4 | 2 | | 2 | 2 | | 483 | | 53.1 | | 7.23 | |  |  |  |
| P36639 | NUDT1 | | 7,8-dihydro-8-oxoguanine triphosphatase OS=Homo sapiens OX=9606 GN=NUDT1 PE=1 SV=3 | | 6 | 1 | | 1 | 1 | | 197 | | 22.5 | | 5.27 | |  |  |  |
| P60709 | ACTB | | Actin, cytoplasmic 1 OS=Homo sapiens OX=9606 GN=ACTB PE=1 SV=1 | | 20 | 7 | | 16 | 7 | | 375 | | 41.7 | | 5.48 | |  |  |  |
| O14639 | ABLIM1 | | Actin-binding LIM protein 1 OS=Homo sapiens OX=9606 GN=ABLIM1 PE=1 SV=3 | | 4 | 2 | | 2 | 2 | | 778 | | 87.6 | | 8.59 | |  |  |  |
| O15143 | ARPC1B | | Actin-related protein 2/3 complex subunit 1B OS=Homo sapiens OX=9606 GN=ARPC1B PE=1 SV=3 | | 3 | 1 | | 1 | 1 | | 372 | | 40.9 | | 8.35 | |  |  |  |
| P30566 | ADSL | | Adenylosuccinate lyase OS=Homo sapiens OX=9606 GN=ADSL PE=1 SV=2 | | 5 | 2 | | 2 | 2 | | 484 | | 54.9 | | 7.11 | |  |  |  |
| Q01518 | CAP1 | | Adenylyl cyclase-associated protein 1 OS=Homo sapiens OX=9606 GN=CAP1 PE=1 SV=5 | | 7 | 3 | | 3 | 2 | | 475 | | 51.9 | | 8.06 | |  |  |  |
| P40123 | CAP2 | | Adenylyl cyclase-associated protein 2 OS=Homo sapiens OX=9606 GN=CAP2 PE=1 SV=1 | | 3 | 2 | | 2 | 1 | | 477 | | 52.8 | | 6.37 | |  |  |  |
| P05141 | SLC25A5 | | ADP/ATP translocase 2 OS=Homo sapiens OX=9606 GN=SLC25A5 PE=1 SV=7 | | 12 | 4 | | 4 | 4 | | 298 | | 32.8 | | 9.69 | |  |  |  |
| P02765 | AHSG | | Alpha-2-HS-glycoprotein OS=Homo sapiens OX=9606 GN=AHSG PE=1 SV=1 | | 3 | 1 | | 1 | 1 | | 367 | | 39.3 | | 5.72 | |  |  |  |
| O43707 | ACTN4 | | Alpha-actinin-4 OS=Homo sapiens OX=9606 GN=ACTN4 PE=1 SV=2 | | 1 | 1 | | 1 | 1 | | 911 | | 104.8 | | 5.44 | |  |  |  |
| P06733 | ENO1 | | Alpha-enolase OS=Homo sapiens OX=9606 GN=ENO1 PE=1 SV=2 | | 2 | 1 | | 1 | 1 | | 434 | | 47.1 | | 7.39 | |  |  |  |
| P07355 | ANXA2 | | Annexin A2 OS=Homo sapiens OX=9606 GN=ANXA2 PE=1 SV=2 | | 13 | 5 | | 5 | 5 | | 339 | | 38.6 | | 7.75 | |  |  |  |
| Q9UKV3 | ACIN1 | | Apoptotic chromatin condensation inducer in the nucleus OS=Homo sapiens OX=9606 GN=ACIN1 PE=1 SV=2 | | 3 | 3 | | 3 | 3 | | 1341 | | 151.8 | | 6.43 | |  |  |  |
| P54136 | RARS | | Arginine--tRNA ligase, cytoplasmic OS=Homo sapiens OX=9606 GN=RARS PE=1 SV=2 | | 1 | 1 | | 1 | 1 | | 660 | | 75.3 | | 6.68 | |  |  |  |
| P14868 | DARS | | Aspartate--tRNA ligase, cytoplasmic OS=Homo sapiens OX=9606 GN=DARS PE=1 SV=2 | | 1 | 1 | | 1 | 1 | | 501 | | 57.1 | | 6.55 | |  |  |  |
| P25705 | ATP5F1A | | ATP synthase subunit alpha, mitochondrial OS=Homo sapiens OX=9606 GN=ATP5F1A PE=1 SV=1 | | 4 | 2 | | 2 | 2 | | 553 | | 59.7 | | 9.13 | |  |  |  |
| P06576 | ATP5F1B | | ATP synthase subunit beta, mitochondrial OS=Homo sapiens OX=9606 GN=ATP5F1B PE=1 SV=3 | | 2 | 1 | | 1 | 1 | | 529 | | 56.5 | | 5.4 | |  |  |  |
| P53396 | ACLY | | ATP-citrate synthase OS=Homo sapiens OX=9606 GN=ACLY PE=1 SV=3 | | 3 | 3 | | 3 | 3 | | 1101 | | 120.8 | | 7.33 | |  |  |  |
| Q08211 | DHX9 | | ATP-dependent RNA helicase A OS=Homo sapiens OX=9606 GN=DHX9 PE=1 SV=4 | | 3 | 4 | | 4 | 4 | | 1270 | | 140.9 | | 6.84 | |  |  |  |
| O00571 | DDX3X | | ATP-dependent RNA helicase DDX3X OS=Homo sapiens OX=9606 GN=DDX3X PE=1 SV=3 | | 3 | 2 | | 2 | 2 | | 662 | | 73.2 | | 7.18 | |  |  |  |
| O95816 | BAG2 | | BAG family molecular chaperone regulator 2 OS=Homo sapiens OX=9606 GN=BAG2 PE=1 SV=1 | | 9 | 2 | | 2 | 2 | | 211 | | 23.8 | | 6.7 | |  |  |  |
| O43491 | EPB41L2 | | Band 4.1-like protein 2 OS=Homo sapiens OX=9606 GN=EPB41L2 PE=1 SV=1 | | 1 | 1 | | 1 | 1 | | 1005 | | 112.5 | | 5.44 | |  |  |  |
| Q9NYF8 | BCLAF1 | | Bcl-2-associated transcription factor 1 OS=Homo sapiens OX=9606 GN=BCLAF1 PE=1 SV=2 | | 7 | 5 | | 6 | 5 | | 920 | | 106.1 | | 9.98 | |  |  |  |
| P07814 | EPRS | | Bifunctional glutamate/proline--tRNA ligase OS=Homo sapiens OX=9606 GN=EPRS PE=1 SV=5 | | 1 | 2 | | 2 | 2 | | 1512 | | 170.5 | | 7.33 | |  |  |  |
| P54132 | BLM | | Bloom syndrome protein OS=Homo sapiens OX=9606 GN=BLM PE=1 SV=1 | | 1 | 1 | | 1 | 1 | | 1417 | | 158.9 | | 7.49 | |  |  |  |
| Q76B58 | BRINP3 | | BMP/retinoic acid-inducible neural-specific protein 3 OS=Homo sapiens OX=9606 GN=BRINP3 PE=1 SV=1 | | 7 | 6 | | 6 | 6 | | 766 | | 88.4 | | 7.81 | |  |  |  |
| P11586 | MTHFD1 | | C-1-tetrahydrofolate synthase, cytoplasmic OS=Homo sapiens OX=9606 GN=MTHFD1 PE=1 SV=3 | | 4 | 4 | | 4 | 4 | | 935 | | 101.5 | | 7.3 | |  |  |  |
| Q13557 | CAMK2D | | Calcium/calmodulin-dependent protein kinase type II subunit delta OS=Homo sapiens OX=9606 GN=CAMK2D PE=1 SV=3 | | 3 | 1 | | 1 | 1 | | 499 | | 56.3 | | 7.25 | |  |  |  |
| Q9UJS0 | SLC25A13 | | Calcium-binding mitochondrial carrier protein Aralar2 OS=Homo sapiens OX=9606 GN=SLC25A13 PE=1 SV=2 | | 1 | 1 | | 1 | 1 | | 675 | | 74.1 | | 8.62 | |  |  |  |
| P27824 | CANX | | Calnexin OS=Homo sapiens OX=9606 GN=CANX PE=1 SV=2 | | 2 | 1 | | 1 | 1 | | 592 | | 67.5 | | 4.6 | |  |  |  |
| P07384 | CAPN1 | | Calpain-1 catalytic subunit OS=Homo sapiens OX=9606 GN=CAPN1 PE=1 SV=1 | | 1 | 1 | | 1 | 1 | | 714 | | 81.8 | | 5.67 | |  |  |  |
| O43852 | CALU | | Calumenin OS=Homo sapiens OX=9606 GN=CALU PE=1 SV=2 | | 2 | 1 | | 1 | 1 | | 315 | | 37.1 | | 4.64 | |  |  |  |
| Q8N163 | CCAR2 | | Cell cycle and apoptosis regulator protein 2 OS=Homo sapiens OX=9606 GN=CCAR2 PE=1 SV=2 | | 1 | 1 | | 1 | 1 | | 923 | | 102.8 | | 5.22 | |  |  |  |
| O00299 | CLIC1 | | Chloride intracellular channel protein 1 OS=Homo sapiens OX=9606 GN=CLIC1 PE=1 SV=4 | | 8 | 2 | | 2 | 2 | | 241 | | 26.9 | | 5.17 | |  |  |  |
| Q9Y696 | CLIC4 | | Chloride intracellular channel protein 4 OS=Homo sapiens OX=9606 GN=CLIC4 PE=1 SV=4 | | 8 | 2 | | 2 | 2 | | 253 | | 28.8 | | 5.59 | |  |  |  |
| Q00610 | CLTC | | Clathrin heavy chain 1 OS=Homo sapiens OX=9606 GN=CLTC PE=1 SV=5 | | 3 | 5 | | 5 | 5 | | 1675 | | 191.5 | | 5.69 | |  |  |  |
| P09496 | CLTA | | Clathrin light chain A OS=Homo sapiens OX=9606 GN=CLTA PE=1 SV=1 | | 4 | 1 | | 1 | 1 | | 248 | | 27.1 | | 4.51 | |  |  |  |
| P23528 | CFL1 | | Cofilin-1 OS=Homo sapiens OX=9606 GN=CFL1 PE=1 SV=3 | | 7 | 1 | | 1 | 1 | | 166 | | 18.5 | | 8.09 | |  |  |  |
| P06493 | CDK1 | | Cyclin-dependent kinase 1 OS=Homo sapiens OX=9606 GN=CDK1 PE=1 SV=3 | | 3 | 1 | | 1 | 1 | | 297 | | 34.1 | | 8.4 | |  |  |  |
| P22695 | UQCRC2 | | Cytochrome b-c1 complex subunit 2, mitochondrial OS=Homo sapiens OX=9606 GN=UQCRC2 PE=1 SV=3 | | 4 | 1 | | 1 | 1 | | 453 | | 48.4 | | 8.63 | |  |  |  |
| P21399 | ACO1 | | Cytoplasmic aconitate hydratase OS=Homo sapiens OX=9606 GN=ACO1 PE=1 SV=3 | | 1 | 1 | | 1 | 1 | | 889 | | 98.3 | | 6.68 | |  |  |  |
| Q14204 | DYNC1H1 | | Cytoplasmic dynein 1 heavy chain 1 OS=Homo sapiens OX=9606 GN=DYNC1H1 PE=1 SV=5 | | 0 | 2 | | 3 | 2 | | 4646 | | 532.1 | | 6.4 | |  |  |  |
| Q07065 | CKAP4 | | Cytoskeleton-associated protein 4 OS=Homo sapiens OX=9606 GN=CKAP4 PE=1 SV=2 | | 4 | 2 | | 2 | 2 | | 602 | | 66 | | 5.92 | |  |  |  |
| Q96KP4 | CNDP2 | | Cytosolic non-specific dipeptidase OS=Homo sapiens OX=9606 GN=CNDP2 PE=1 SV=2 | | 2 | 1 | | 1 | 1 | | 475 | | 52.8 | | 5.97 | |  |  |  |
| O43175 | PHGDH | | D-3-phosphoglycerate dehydrogenase OS=Homo sapiens OX=9606 GN=PHGDH PE=1 SV=4 | | 3 | 1 | | 1 | 1 | | 533 | | 56.6 | | 6.71 | |  |  |  |
| Q13268 | DHRS2 | | Dehydrogenase/reductase SDR family member 2, mitochondrial OS=Homo sapiens OX=9606 GN=DHRS2 PE=1 SV=4 | | 4 | 1 | | 1 | 1 | | 280 | | 29.9 | | 9.01 | |  |  |  |
| P09622 | DLD | | Dihydrolipoyl dehydrogenase, mitochondrial OS=Homo sapiens OX=9606 GN=DLD PE=1 SV=2 | | 2 | 1 | | 1 | 1 | | 509 | | 54.1 | | 7.85 | |  |  |  |
| Q16531 | DDB1 | | DNA damage-binding protein 1 OS=Homo sapiens OX=9606 GN=DDB1 PE=1 SV=1 | | 3 | 3 | | 3 | 3 | | 1140 | | 126.9 | | 5.26 | |  |  |  |
| P49736 | MCM2 | | DNA replication licensing factor MCM2 OS=Homo sapiens OX=9606 GN=MCM2 PE=1 SV=4 | | 1 | 1 | | 1 | 1 | | 904 | | 101.8 | | 5.52 | |  |  |  |
| P33993 | MCM7 | | DNA replication licensing factor MCM7 OS=Homo sapiens OX=9606 GN=MCM7 PE=1 SV=4 | | 1 | 1 | | 1 | 1 | | 719 | | 81.3 | | 6.46 | |  |  |  |
| P04843 | RPN1 | | Dolichyl-diphosphooligosaccharide--protein glycosyltransferase subunit 1 OS=Homo sapiens OX=9606 GN=RPN1 PE=1 SV=1 | | 3 | 2 | | 2 | 2 | | 607 | | 68.5 | | 6.38 | |  |  |  |
| Q16643 | DBN1 | | Drebrin OS=Homo sapiens OX=9606 GN=DBN1 PE=1 SV=4 | | 5 | 2 | | 3 | 2 | | 649 | | 71.4 | | 4.45 | |  |  |  |
| Q14203 | DCTN1 | | Dynactin subunit 1 OS=Homo sapiens OX=9606 GN=DCTN1 PE=1 SV=3 | | 1 | 1 | | 1 | 1 | | 1278 | | 141.6 | | 5.81 | |  |  |  |
| Q13561 | DCTN2 | | Dynactin subunit 2 OS=Homo sapiens OX=9606 GN=DCTN2 PE=1 SV=4 | | 2 | 1 | | 1 | 1 | | 401 | | 44.2 | | 5.21 | |  |  |  |
| Q14258 | TRIM25 | | E3 ubiquitin/ISG15 ligase TRIM25 OS=Homo sapiens OX=9606 GN=TRIM25 PE=1 SV=2 | | 2 | 1 | | 1 | 1 | | 630 | | 70.9 | | 8.09 | |  |  |  |
| P19474 | TRIM21 | | E3 ubiquitin-protein ligase TRIM21 OS=Homo sapiens OX=9606 GN=TRIM21 PE=1 SV=1 | | 5 | 2 | | 2 | 2 | | 475 | | 54.1 | | 6.38 | |  |  |  |
| Q05639 | EEF1A2 | | Elongation factor 1-alpha 2 OS=Homo sapiens OX=9606 GN=EEF1A2 PE=1 SV=1 | | 6 | 3 | | 4 | 3 | | 463 | | 50.4 | | 9.03 | |  |  |  |
| P26641 | EEF1G | | Elongation factor 1-gamma OS=Homo sapiens OX=9606 GN=EEF1G PE=1 SV=3 | | 3 | 1 | | 1 | 1 | | 437 | | 50.1 | | 6.67 | |  |  |  |
| P13639 | EEF2 | | Elongation factor 2 OS=Homo sapiens OX=9606 GN=EEF2 PE=1 SV=4 | | 3 | 3 | | 3 | 3 | | 858 | | 95.3 | | 6.83 | |  |  |  |
| P11021 | HSPA5 | | Endoplasmic reticulum chaperone BiP OS=Homo sapiens OX=9606 GN=HSPA5 PE=1 SV=2 | | 6 | 4 | | 4 | 3 | | 654 | | 72.3 | | 5.16 | |  |  |  |
| P14625 | HSP90B1 | | Endoplasmin OS=Homo sapiens OX=9606 GN=HSP90B1 PE=1 SV=1 | | 5 | 5 | | 5 | 4 | | 803 | | 92.4 | | 4.84 | |  |  |  |
| P84090 | ERH | | Enhancer of rudimentary homolog OS=Homo sapiens OX=9606 GN=ERH PE=1 SV=1 | | 11 | 1 | | 1 | 1 | | 104 | | 12.3 | | 5.92 | |  |  |  |
| O75477 | ERLIN1 | | Erlin-1 OS=Homo sapiens OX=9606 GN=ERLIN1 PE=1 SV=1 | | 4 | 1 | | 1 | 1 | | 346 | | 38.9 | | 7.87 | |  |  |  |
| P60842 | EIF4A1 | | Eukaryotic initiation factor 4A-I OS=Homo sapiens OX=9606 GN=EIF4A1 PE=1 SV=1 | | 3 | 1 | | 1 | 1 | | 406 | | 46.1 | | 5.48 | |  |  |  |
| P38919 | EIF4A3 | | Eukaryotic initiation factor 4A-III OS=Homo sapiens OX=9606 GN=EIF4A3 PE=1 SV=4 | | 3 | 1 | | 1 | 1 | | 411 | | 46.8 | | 6.73 | |  |  |  |
| P05198 | EIF2S1 | | Eukaryotic translation initiation factor 2 subunit 1 OS=Homo sapiens OX=9606 GN=EIF2S1 PE=1 SV=3 | | 5 | 2 | | 2 | 2 | | 315 | | 36.1 | | 5.08 | |  |  |  |
| P41091 | EIF2S3 | | Eukaryotic translation initiation factor 2 subunit 3 OS=Homo sapiens OX=9606 GN=EIF2S3 PE=1 SV=3 | | 2 | 1 | | 1 | 1 | | 472 | | 51.1 | | 8.4 | |  |  |  |
| Q14152 | EIF3A | | Eukaryotic translation initiation factor 3 subunit A OS=Homo sapiens OX=9606 GN=EIF3A PE=1 SV=1 | | 1 | 1 | | 1 | 1 | | 1382 | | 166.5 | | 6.79 | |  |  |  |
| P55884 | EIF3B | | Eukaryotic translation initiation factor 3 subunit B OS=Homo sapiens OX=9606 GN=EIF3B PE=1 SV=3 | | 2 | 1 | | 1 | 1 | | 814 | | 92.4 | | 5 | |  |  |  |
| B5ME19 | EIF3CL | | Eukaryotic translation initiation factor 3 subunit C-like protein OS=Homo sapiens OX=9606 GN=EIF3CL PE=3 SV=1 | | 1 | 1 | | 1 | 1 | | 914 | | 105.4 | | 5.64 | |  |  |  |
| O00303 | EIF3F | | Eukaryotic translation initiation factor 3 subunit F OS=Homo sapiens OX=9606 GN=EIF3F PE=1 SV=1 | | 3 | 1 | | 1 | 1 | | 357 | | 37.5 | | 5.45 | |  |  |  |
| P78344 | EIF4G2 | | Eukaryotic translation initiation factor 4 gamma 2 OS=Homo sapiens OX=9606 GN=EIF4G2 PE=1 SV=1 | | 2 | 2 | | 2 | 2 | | 907 | | 102.3 | | 7.14 | |  |  |  |
| P23588 | EIF4B | | Eukaryotic translation initiation factor 4B OS=Homo sapiens OX=9606 GN=EIF4B PE=1 SV=2 | | 33 | 21 | | 38 | 21 | | 611 | | 69.1 | | 5.73 | |  |  |  |
| P15311 | EZR | | Ezrin OS=Homo sapiens OX=9606 GN=EZR PE=1 SV=4 | | 1 | 1 | | 1 | 1 | | 586 | | 69.4 | | 6.27 | |  |  |  |
| Q9Y5B9 | SUPT16H | | FACT complex subunit SPT16 OS=Homo sapiens OX=9606 GN=SUPT16H PE=1 SV=1 | | 1 | 1 | | 1 | 1 | | 1047 | | 119.8 | | 5.66 | |  |  |  |
| P52907 | CAPZA1 | | F-actin-capping protein subunit alpha-1 OS=Homo sapiens OX=9606 GN=CAPZA1 PE=1 SV=3 | | 5 | 1 | | 1 | 1 | | 286 | | 32.9 | | 5.69 | |  |  |  |
| Q16658 | FSCN1 | | Fascin OS=Homo sapiens OX=9606 GN=FSCN1 PE=1 SV=3 | | 5 | 2 | | 2 | 2 | | 493 | | 54.5 | | 7.24 | |  |  |  |
| P49327 | FASN | | Fatty acid synthase OS=Homo sapiens OX=9606 GN=FASN PE=1 SV=3 | | 2 | 6 | | 6 | 6 | | 2511 | | 273.3 | | 6.44 | |  |  |  |
| Q96AC1 | FERMT2 | | Fermitin family homolog 2 OS=Homo sapiens OX=9606 GN=FERMT2 PE=1 SV=1 | | 2 | 1 | | 1 | 1 | | 680 | | 77.8 | | 6.7 | |  |  |  |
| P21333 | FLNA | | Filamin-A OS=Homo sapiens OX=9606 GN=FLNA PE=1 SV=4 | | 12 | 26 | | 32 | 24 | | 2647 | | 280.6 | | 6.06 | |  |  |  |
| O75369 | FLNB | | Filamin-B OS=Homo sapiens OX=9606 GN=FLNB PE=1 SV=2 | | 1 | 2 | | 2 | 2 | | 2602 | | 278 | | 5.73 | |  |  |  |
| Q14315 | FLNC | | Filamin-C OS=Homo sapiens OX=9606 GN=FLNC PE=1 SV=3 | | 4 | 11 | | 11 | 9 | | 2725 | | 290.8 | | 5.97 | |  |  |  |
| P04075 | ALDOA | | Fructose-bisphosphate aldolase A OS=Homo sapiens OX=9606 GN=ALDOA PE=1 SV=2 | | 5 | 2 | | 2 | 2 | | 364 | | 39.4 | | 8.09 | |  |  |  |
| P06396 | GSN | | Gelsolin OS=Homo sapiens OX=9606 GN=GSN PE=1 SV=1 | | 2 | 2 | | 2 | 2 | | 782 | | 85.6 | | 6.28 | |  |  |  |
| P11413 | G6PD | | Glucose-6-phosphate 1-dehydrogenase OS=Homo sapiens OX=9606 GN=G6PD PE=1 SV=4 | | 3 | 2 | | 2 | 2 | | 515 | | 59.2 | | 6.84 | |  |  |  |
| P06744 | GPI | | Glucose-6-phosphate isomerase OS=Homo sapiens OX=9606 GN=GPI PE=1 SV=4 | | 2 | 1 | | 1 | 1 | | 558 | | 63.1 | | 8.32 | |  |  |  |
| P04406 | GAPDH | | Glyceraldehyde-3-phosphate dehydrogenase OS=Homo sapiens OX=9606 GN=GAPDH PE=1 SV=3 | | 17 | 6 | | 8 | 6 | | 335 | | 36 | | 8.46 | |  |  |  |
| P62826 | RAN | | GTP-binding nuclear protein Ran OS=Homo sapiens OX=9606 GN=RAN PE=1 SV=3 | | 10 | 2 | | 2 | 2 | | 216 | | 24.4 | | 7.49 | |  |  |  |
| P0DMV8 | HSPA1A | | Heat shock 70 kDa protein 1A OS=Homo sapiens OX=9606 GN=HSPA1A PE=1 SV=1 | | 5 | 4 | | 4 | 1 | | 641 | | 70 | | 5.66 | |  |  |  |
| P11142 | HSPA8 | | Heat shock cognate 71 kDa protein OS=Homo sapiens OX=9606 GN=HSPA8 PE=1 SV=1 | | 12 | 8 | | 9 | 5 | | 646 | | 70.9 | | 5.52 | |  |  |  |
| P04792 | HSPB1 | | Heat shock protein beta-1 OS=Homo sapiens OX=9606 GN=HSPB1 PE=1 SV=2 | | 9 | 3 | | 3 | 3 | | 205 | | 22.8 | | 6.4 | |  |  |  |
| P07900 | HSP90AA1 | | Heat shock protein HSP 90-alpha OS=Homo sapiens OX=9606 GN=HSP90AA1 PE=1 SV=5 | | 13 | 12 | | 15 | 4 | | 732 | | 84.6 | | 5.02 | |  |  |  |
| P08238 | HSP90AB1 | | Heat shock protein HSP 90-beta OS=Homo sapiens OX=9606 GN=HSP90AB1 PE=1 SV=4 | | 14 | 12 | | 15 | 3 | | 724 | | 83.2 | | 5.03 | |  |  |  |
| Q99729 | HNRNPAB | | Heterogeneous nuclear ribonucleoprotein A/B OS=Homo sapiens OX=9606 GN=HNRNPAB PE=1 SV=2 | | 7 | 2 | | 2 | 1 | | 332 | | 36.2 | | 8.21 | |  |  |  |
| P09651 | HNRNPA1 | | Heterogeneous nuclear ribonucleoprotein A1 OS=Homo sapiens OX=9606 GN=HNRNPA1 PE=1 SV=5 | | 14 | 4 | | 4 | 4 | | 372 | | 38.7 | | 9.13 | |  |  |  |
| Q14103 | HNRNPD | | Heterogeneous nuclear ribonucleoprotein D0 OS=Homo sapiens OX=9606 GN=HNRNPD PE=1 SV=1 | | 7 | 2 | | 2 | 1 | | 355 | | 38.4 | | 7.81 | |  |  |  |
| P31943 | HNRNPH1 | | Heterogeneous nuclear ribonucleoprotein H OS=Homo sapiens OX=9606 GN=HNRNPH1 PE=1 SV=4 | | 6 | 2 | | 2 | 2 | | 449 | | 49.2 | | 6.3 | |  |  |  |
| P61978 | HNRNPK | | Heterogeneous nuclear ribonucleoprotein K OS=Homo sapiens OX=9606 GN=HNRNPK PE=1 SV=1 | | 8 | 3 | | 3 | 3 | | 463 | | 50.9 | | 5.54 | |  |  |  |
| P52272 | HNRNPM | | Heterogeneous nuclear ribonucleoprotein M OS=Homo sapiens OX=9606 GN=HNRNPM PE=1 SV=3 | | 2 | 2 | | 2 | 2 | | 730 | | 77.5 | | 8.7 | |  |  |  |
| Q00839 | HNRNPU | | Heterogeneous nuclear ribonucleoprotein U OS=Homo sapiens OX=9606 GN=HNRNPU PE=1 SV=6 | | 1 | 1 | | 1 | 1 | | 825 | | 90.5 | | 6 | |  |  |  |
| P07910 | HNRNPC | | Heterogeneous nuclear ribonucleoproteins C1/C2 OS=Homo sapiens OX=9606 GN=HNRNPC PE=1 SV=4 | | 3 | 1 | | 1 | 1 | | 306 | | 33.7 | | 5.08 | |  |  |  |
| O00422 | SAP18 | | Histone deacetylase complex subunit SAP18 OS=Homo sapiens OX=9606 GN=SAP18 PE=1 SV=1 | | 5 | 1 | | 1 | 1 | | 153 | | 17.6 | | 9.35 | |  |  |  |
| P16403 | HIST1H1C | | Histone H1.2 OS=Homo sapiens OX=9606 GN=HIST1H1C PE=1 SV=2 | | 23 | 5 | | 6 | 1 | | 213 | | 21.4 | | 10.93 | |  |  |  |
| P10412 | HIST1H1E | | Histone H1.4 OS=Homo sapiens OX=9606 GN=HIST1H1E PE=1 SV=2 | | 22 | 5 | | 6 | 1 | | 219 | | 21.9 | | 11.03 | |  |  |  |
| P16104 | H2AFX | | Histone H2AX OS=Homo sapiens OX=9606 GN=H2AFX PE=1 SV=2 | | 6 | 1 | | 1 | 1 | | 143 | | 15.1 | | 10.74 | |  |  |  |
| O60814 | HIST1H2BK | | Histone H2B type 1-K OS=Homo sapiens OX=9606 GN=HIST1H2BK PE=1 SV=3 | | 14 | 2 | | 2 | 2 | | 126 | | 13.9 | | 10.32 | |  |  |  |
| P68431 | HIST1H3A | | Histone H3.1 OS=Homo sapiens OX=9606 GN=HIST1H3A PE=1 SV=2 | | 5 | 1 | | 1 | 1 | | 136 | | 15.4 | | 11.12 | |  |  |  |
| P62805 | HIST1H4A | | Histone H4 OS=Homo sapiens OX=9606 GN=HIST1H4A PE=1 SV=2 | | 31 | 3 | | 4 | 3 | | 103 | | 11.4 | | 11.36 | |  |  |  |
| Q86YZ3 | HRNR | | Hornerin OS=Homo sapiens OX=9606 GN=HRNR PE=1 SV=2 | | 3 | 1 | | 1 | 1 | | 2850 | | 282.2 | | 10.04 | |  |  |  |
| Q01581 | HMGCS1 | | Hydroxymethylglutaryl-CoA synthase, cytoplasmic OS=Homo sapiens OX=9606 GN=HMGCS1 PE=1 SV=2 | | 3 | 1 | | 1 | 1 | | 520 | | 57.3 | | 5.41 | |  |  |  |
| P0DOX5 |  | | Immunoglobulin gamma-1 heavy chain OS=Homo sapiens OX=9606 PE=1 SV=2 | | 2 | 1 | | 1 | 1 | | 449 | | 49.3 | | 8.72 | |  |  |  |
| P01859 | IGHG2 | | Immunoglobulin heavy constant gamma 2 OS=Homo sapiens OX=9606 GN=IGHG2 PE=1 SV=2 | | 3 | 1 | | 1 | 1 | | 326 | | 35.9 | | 7.59 | |  |  |  |
| P01614 | IGKV2D-40 | | Immunoglobulin kappa variable 2D-40 OS=Homo sapiens OX=9606 GN=IGKV2D-40 PE=1 SV=2 | | 11 | 1 | | 3 | 1 | | 121 | | 13.3 | | 4.61 | |  |  |  |
| P20839 | IMPDH1 | | Inosine-5'-monophosphate dehydrogenase 1 OS=Homo sapiens OX=9606 GN=IMPDH1 PE=1 SV=2 | | 4 | 2 | | 2 | 1 | | 514 | | 55.4 | | 6.9 | |  |  |  |
| P12268 | IMPDH2 | | Inosine-5'-monophosphate dehydrogenase 2 OS=Homo sapiens OX=9606 GN=IMPDH2 PE=1 SV=2 | | 10 | 6 | | 7 | 5 | | 514 | | 55.8 | | 6.9 | |  |  |  |
| Q13418 | ILK | | Integrin-linked protein kinase OS=Homo sapiens OX=9606 GN=ILK PE=1 SV=2 | | 2 | 1 | | 1 | 1 | | 452 | | 51.4 | | 8.07 | |  |  |  |
| Q12906 | ILF3 | | Interleukin enhancer-binding factor 3 OS=Homo sapiens OX=9606 GN=ILF3 PE=1 SV=3 | | 1 | 1 | | 1 | 1 | | 894 | | 95.3 | | 8.76 | |  |  |  |
| P20700 | LMNB1 | | Lamin-B1 OS=Homo sapiens OX=9606 GN=LMNB1 PE=1 SV=2 | | 5 | 3 | | 3 | 2 | | 586 | | 66.4 | | 5.16 | |  |  |  |
| Q14766 | LTBP1 | | Latent-transforming growth factor beta-binding protein 1 OS=Homo sapiens OX=9606 GN=LTBP1 PE=1 SV=4 | | 1 | 1 | | 1 | 1 | | 1721 | | 186.7 | | 5.96 | |  |  |  |
| P42704 | LRPPRC | | Leucine-rich PPR motif-containing protein, mitochondrial OS=Homo sapiens OX=9606 GN=LRPPRC PE=1 SV=3 | | 1 | 1 | | 1 | 1 | | 1394 | | 157.8 | | 6.13 | |  |  |  |
| Q9P2J5 | LARS | | Leucine--tRNA ligase, cytoplasmic OS=Homo sapiens OX=9606 GN=LARS PE=1 SV=2 | | 1 | 1 | | 1 | 1 | | 1176 | | 134.4 | | 7.3 | |  |  |  |
| P09960 | LTA4H | | Leukotriene A-4 hydrolase OS=Homo sapiens OX=9606 GN=LTA4H PE=1 SV=2 | | 2 | 1 | | 1 | 1 | | 611 | | 69.2 | | 6.18 | |  |  |  |
| Q9UHB6 | LIMA1 | | LIM domain and actin-binding protein 1 OS=Homo sapiens OX=9606 GN=LIMA1 PE=1 SV=1 | | 1 | 1 | | 1 | 1 | | 759 | | 85.2 | | 6.84 | |  |  |  |
| P00338 | LDHA | | L-lactate dehydrogenase A chain OS=Homo sapiens OX=9606 GN=LDHA PE=1 SV=2 | | 12 | 4 | | 4 | 3 | | 332 | | 36.7 | | 8.27 | |  |  |  |
| P07195 | LDHB | | L-lactate dehydrogenase B chain OS=Homo sapiens OX=9606 GN=LDHB PE=1 SV=2 | | 14 | 5 | | 7 | 4 | | 334 | | 36.6 | | 6.05 | |  |  |  |
| P43361 | MAGEA8 | | Melanoma-associated antigen 8 OS=Homo sapiens OX=9606 GN=MAGEA8 PE=1 SV=2 | | 5 | 2 | | 2 | 2 | | 318 | | 35.2 | | 4.77 | |  |  |  |
| Q9UNF1 | MAGED2 | | Melanoma-associated antigen D2 OS=Homo sapiens OX=9606 GN=MAGED2 PE=1 SV=2 | | 3 | 2 | | 2 | 2 | | 606 | | 64.9 | | 9.32 | |  |  |  |
| P56192 | MARS | | Methionine--tRNA ligase, cytoplasmic OS=Homo sapiens OX=9606 GN=MARS PE=1 SV=2 | | 1 | 1 | | 1 | 1 | | 900 | | 101.1 | | 6.16 | |  |  |  |
| Q9BQA1 | WDR77 | | Methylosome protein 50 OS=Homo sapiens OX=9606 GN=WDR77 PE=1 SV=1 | | 10 | 3 | | 3 | 3 | | 342 | | 36.7 | | 5.17 | |  |  |  |
| P27816 | MAP4 | | Microtubule-associated protein 4 OS=Homo sapiens OX=9606 GN=MAP4 PE=1 SV=3 | | 3 | 2 | | 2 | 2 | | 1152 | | 120.9 | | 5.43 | |  |  |  |
| Q6P1R3 | MSANTD2 | | Myb/SANT-like DNA-binding domain-containing protein 2 OS=Homo sapiens OX=9606 GN=MSANTD2 PE=1 SV=1 | | 2 | 1 | | 1 | 1 | | 559 | | 61.3 | | 6.19 | |  |  |  |
| P60660 | MYL6 | | Myosin light polypeptide 6 OS=Homo sapiens OX=9606 GN=MYL6 PE=1 SV=2 | | 15 | 2 | | 2 | 2 | | 151 | | 16.9 | | 4.65 | |  |  |  |
| P35579 | MYH9 | | Myosin-9 OS=Homo sapiens OX=9606 GN=MYH9 PE=1 SV=4 | | 7 | 12 | | 13 | 12 | | 1960 | | 226.4 | | 5.6 | |  |  |  |
| Q13423 | NNT | | NAD(P) transhydrogenase, mitochondrial OS=Homo sapiens OX=9606 GN=NNT PE=1 SV=3 | | 1 | 1 | | 1 | 1 | | 1086 | | 113.8 | | 8.09 | |  |  |  |
| P16435 | POR | | NADPH--cytochrome P450 reductase OS=Homo sapiens OX=9606 GN=POR PE=1 SV=2 | | 1 | 1 | | 1 | 1 | | 677 | | 76.6 | | 5.58 | |  |  |  |
| Q09666 | AHNAK | | Neuroblast differentiation-associated protein AHNAK OS=Homo sapiens OX=9606 GN=AHNAK PE=1 SV=2 | | 2 | 3 | | 4 | 3 | | 5890 | | 628.7 | | 6.15 | |  |  |  |
| Q14697 | GANAB | | Neutral alpha-glucosidase AB OS=Homo sapiens OX=9606 GN=GANAB PE=1 SV=3 | | 2 | 2 | | 2 | 2 | | 944 | | 106.8 | | 6.14 | |  |  |  |
| Q0ZGT2 | NEXN | | Nexilin OS=Homo sapiens OX=9606 GN=NEXN PE=1 SV=1 | | 2 | 1 | | 1 | 1 | | 675 | | 80.6 | | 5.33 | |  |  |  |
| P43490 | NAMPT | | Nicotinamide phosphoribosyltransferase OS=Homo sapiens OX=9606 GN=NAMPT PE=1 SV=1 | | 1 | 1 | | 1 | 1 | | 491 | | 55.5 | | 7.15 | |  |  |  |
| P49321 | NASP | | Nuclear autoantigenic sperm protein OS=Homo sapiens OX=9606 GN=NASP PE=1 SV=2 | | 1 | 1 | | 1 | 1 | | 788 | | 85.2 | | 4.3 | |  |  |  |
| P67809 | YBX1 | | Nuclease-sensitive element-binding protein 1 OS=Homo sapiens OX=9606 GN=YBX1 PE=1 SV=3 | | 19 | 4 | | 4 | 3 | | 324 | | 35.9 | | 9.88 | |  |  |  |
| Q9NR30 | DDX21 | | Nucleolar RNA helicase 2 OS=Homo sapiens OX=9606 GN=DDX21 PE=1 SV=5 | | 2 | 1 | | 1 | 1 | | 783 | | 87.3 | | 9.28 | |  |  |  |
| P19338 | NCL | | Nucleolin OS=Homo sapiens OX=9606 GN=NCL PE=1 SV=3 | | 5 | 4 | | 4 | 4 | | 710 | | 76.6 | | 4.7 | |  |  |  |
| P06748 | NPM1 | | Nucleophosmin OS=Homo sapiens OX=9606 GN=NPM1 PE=1 SV=2 | | 10 | 3 | | 4 | 3 | | 294 | | 32.6 | | 4.78 | |  |  |  |
| Q9Y5B8 | NME7 | | Nucleoside diphosphate kinase 7 OS=Homo sapiens OX=9606 GN=NME7 PE=1 SV=1 | | 3 | 1 | | 1 | 1 | | 376 | | 42.5 | | 6.47 | |  |  |  |
| Q8WX93 | PALLD | | Palladin OS=Homo sapiens OX=9606 GN=PALLD PE=1 SV=3 | | 1 | 1 | | 1 | 1 | | 1383 | | 150.5 | | 7.09 | |  |  |  |
| Q06830 | PRDX1 | | Peroxiredoxin-1 OS=Homo sapiens OX=9606 GN=PRDX1 PE=1 SV=1 | | 14 | 3 | | 3 | 3 | | 199 | | 22.1 | | 8.13 | |  |  |  |
| P30041 | PRDX6 | | Peroxiredoxin-6 OS=Homo sapiens OX=9606 GN=PRDX6 PE=1 SV=3 | | 16 | 3 | | 3 | 3 | | 224 | | 25 | | 6.38 | |  |  |  |
| O00541 | PES1 | | Pescadillo homolog OS=Homo sapiens OX=9606 GN=PES1 PE=1 SV=1 | | 1 | 1 | | 1 | 1 | | 588 | | 68 | | 7.33 | |  |  |  |
| Q9Y263 | PLAA | | Phospholipase A-2-activating protein OS=Homo sapiens OX=9606 GN=PLAA PE=1 SV=2 | | 2 | 1 | | 1 | 1 | | 795 | | 87.1 | | 6.37 | |  |  |  |
| Q9Y617 | PSAT1 | | Phosphoserine aminotransferase OS=Homo sapiens OX=9606 GN=PSAT1 PE=1 SV=2 | | 3 | 1 | | 1 | 1 | | 370 | | 40.4 | | 7.66 | |  |  |  |
| Q15149 | PLEC | | Plectin OS=Homo sapiens OX=9606 GN=PLEC PE=1 SV=3 | | 2 | 8 | | 8 | 8 | | 4684 | | 531.5 | | 5.96 | |  |  |  |
| P09874 | PARP1 | | Poly [ADP-ribose] polymerase 1 OS=Homo sapiens OX=9606 GN=PARP1 PE=1 SV=4 | | 1 | 1 | | 1 | 1 | | 1014 | | 113 | | 8.88 | |  |  |  |
| Q15365 | PCBP1 | | Poly(rC)-binding protein 1 OS=Homo sapiens OX=9606 GN=PCBP1 PE=1 SV=2 | | 8 | 3 | | 3 | 3 | | 356 | | 37.5 | | 7.09 | |  |  |  |
| P11940 | PABPC1 | | Polyadenylate-binding protein 1 OS=Homo sapiens OX=9606 GN=PABPC1 PE=1 SV=2 | | 3 | 2 | | 2 | 2 | | 636 | | 70.6 | | 9.5 | |  |  |  |
| P02545 | LMNA | | Prelamin-A/C OS=Homo sapiens OX=9606 GN=LMNA PE=1 SV=1 | | 3 | 2 | | 2 | 1 | | 664 | | 74.1 | | 7.02 | |  |  |  |
| Q92841 | DDX17 | | Probable ATP-dependent RNA helicase DDX17 OS=Homo sapiens OX=9606 GN=DDX17 PE=1 SV=2 | | 2 | 1 | | 1 | 1 | | 729 | | 80.2 | | 8.27 | |  |  |  |
| Q8WUM4 | PDCD6IP | | Programmed cell death 6-interacting protein OS=Homo sapiens OX=9606 GN=PDCD6IP PE=1 SV=1 | | 1 | 1 | | 1 | 1 | | 868 | | 96 | | 6.52 | |  |  |  |
| P12004 | PCNA | | Proliferating cell nuclear antigen OS=Homo sapiens OX=9606 GN=PCNA PE=1 SV=1 | | 5 | 1 | | 1 | 1 | | 261 | | 28.8 | | 4.69 | |  |  |  |
| Q32P28 | P3H1 | | Prolyl 3-hydroxylase 1 OS=Homo sapiens OX=9606 GN=P3H1 PE=1 SV=2 | | 3 | 2 | | 2 | 2 | | 736 | | 83.3 | | 5.14 | |  |  |  |
| P25789 | PSMA4 | | Proteasome subunit alpha type-4 OS=Homo sapiens OX=9606 GN=PSMA4 PE=1 SV=1 | | 3 | 1 | | 1 | 1 | | 261 | | 29.5 | | 7.72 | |  |  |  |
| O14744 | PRMT5 | | Protein arginine N-methyltransferase 5 OS=Homo sapiens OX=9606 GN=PRMT5 PE=1 SV=4 | | 14 | 9 | | 9 | 9 | | 637 | | 72.6 | | 6.29 | |  |  |  |
| P30101 | PDIA3 | | Protein disulfide-isomerase A3 OS=Homo sapiens OX=9606 GN=PDIA3 PE=1 SV=4 | | 3 | 1 | | 1 | 1 | | 505 | | 56.7 | | 6.35 | |  |  |  |
| Q15084 | PDIA6 | | Protein disulfide-isomerase A6 OS=Homo sapiens OX=9606 GN=PDIA6 PE=1 SV=1 | | 3 | 1 | | 1 | 1 | | 440 | | 48.1 | | 5.08 | |  |  |  |
| P07237 | P4HB | | Protein disulfide-isomerase OS=Homo sapiens OX=9606 GN=P4HB PE=1 SV=3 | | 2 | 1 | | 1 | 1 | | 508 | | 57.1 | | 4.87 | |  |  |  |
| O75688 | PPM1B | | Protein phosphatase 1B OS=Homo sapiens OX=9606 GN=PPM1B PE=1 SV=1 | | 4 | 2 | | 3 | 2 | | 479 | | 52.6 | | 5.05 | |  |  |  |
| P53992 | SEC24C | | Protein transport protein Sec24C OS=Homo sapiens OX=9606 GN=SEC24C PE=1 SV=3 | | 1 | 1 | | 1 | 1 | | 1094 | | 118.2 | | 7.06 | |  |  |  |
| Q96MG8 | PCMTD1 | | Protein-L-isoaspartate O-methyltransferase domain-containing protein 1 OS=Homo sapiens OX=9606 GN=PCMTD1 PE=1 SV=2 | | 2 | 1 | | 1 | 1 | | 357 | | 40.7 | | 5.66 | |  |  |  |
| P14618 | PKM | | Pyruvate kinase PKM OS=Homo sapiens OX=9606 GN=PKM PE=1 SV=4 | | 4 | 2 | | 2 | 2 | | 531 | | 57.9 | | 7.84 | |  |  |  |
| P63244 | RACK1 | | Receptor of activated protein C kinase 1 OS=Homo sapiens OX=9606 GN=RACK1 PE=1 SV=3 | | 3 | 1 | | 1 | 1 | | 317 | | 35.1 | | 7.69 | |  |  |  |
| Q13464 | ROCK1 | | Rho-associated protein kinase 1 OS=Homo sapiens OX=9606 GN=ROCK1 PE=1 SV=1 | | 1 | 1 | | 1 | 1 | | 1354 | | 158.1 | | 5.9 | |  |  |  |
| P13489 | RNH1 | | Ribonuclease inhibitor OS=Homo sapiens OX=9606 GN=RNH1 PE=1 SV=2 | | 2 | 1 | | 1 | 1 | | 461 | | 49.9 | | 4.82 | |  |  |  |
| P98175 | RBM10 | | RNA-binding protein 10 OS=Homo sapiens OX=9606 GN=RBM10 PE=1 SV=3 | | 5 | 4 | | 4 | 4 | | 930 | | 103.5 | | 5.97 | |  |  |  |
| P34897 | SHMT2 | | Serine hydroxymethyltransferase, mitochondrial OS=Homo sapiens OX=9606 GN=SHMT2 PE=1 SV=3 | | 2 | 1 | | 1 | 1 | | 504 | | 56 | | 8.53 | |  |  |  |
| Q07955 | SRSF1 | | Serine/arginine-rich splicing factor 1 OS=Homo sapiens OX=9606 GN=SRSF1 PE=1 SV=2 | | 4 | 1 | | 1 | 1 | | 248 | | 27.7 | | 10.36 | |  |  |  |
| Q13247 | SRSF6 | | Serine/arginine-rich splicing factor 6 OS=Homo sapiens OX=9606 GN=SRSF6 PE=1 SV=2 | | 5 | 2 | | 2 | 2 | | 344 | | 39.6 | | 11.43 | |  |  |  |
| Q16629 | SRSF7 | | Serine/arginine-rich splicing factor 7 OS=Homo sapiens OX=9606 GN=SRSF7 PE=1 SV=1 | | 5 | 1 | | 1 | 1 | | 238 | | 27.4 | | 11.82 | |  |  |  |
| Q9BRL6 | SRSF8 | | Serine/arginine-rich splicing factor 8 OS=Homo sapiens OX=9606 GN=SRSF8 PE=1 SV=1 | | 5 | 2 | | 2 | 2 | | 282 | | 32.3 | | 11.72 | |  |  |  |
| P62136 | PPP1CA | | Serine/threonine-protein phosphatase PP1-alpha catalytic subunit OS=Homo sapiens OX=9606 GN=PPP1CA PE=1 SV=1 | | 3 | 1 | | 1 | 1 | | 330 | | 37.5 | | 6.33 | |  |  |  |
| P50454 | SERPINH1 | | Serpin H1 OS=Homo sapiens OX=9606 GN=SERPINH1 PE=1 SV=2 | | 3 | 1 | | 1 | 1 | | 418 | | 46.4 | | 8.69 | |  |  |  |
| Q9BXP5 | SRRT | | Serrate RNA effector molecule homolog OS=Homo sapiens OX=9606 GN=SRRT PE=1 SV=1 | | 1 | 1 | | 1 | 1 | | 876 | | 100.6 | | 5.96 | |  |  |  |
| P02768 | ALB | | Serum albumin OS=Homo sapiens OX=9606 GN=ALB PE=1 SV=2 | | 16 | 10 | | 11 | 10 | | 609 | | 69.3 | | 6.28 | |  |  |  |
| Q9Y5M8 | SRPRB | | Signal recognition particle receptor subunit beta OS=Homo sapiens OX=9606 GN=SRPRB PE=1 SV=3 | | 7 | 1 | | 1 | 1 | | 271 | | 29.7 | | 9.04 | |  |  |  |
| Q9P270 | SLAIN2 | | SLAIN motif-containing protein 2 OS=Homo sapiens OX=9606 GN=SLAIN2 PE=1 SV=2 | | 3 | 1 | | 1 | 1 | | 581 | | 62.5 | | 9.45 | |  |  |  |
| P55854 | SUMO3 | | Small ubiquitin-related modifier 3 OS=Homo sapiens OX=9606 GN=SUMO3 PE=1 SV=2 | | 12 | 1 | | 1 | 1 | | 103 | | 11.6 | | 5.49 | |  |  |  |
| P05023 | ATP1A1 | | Sodium/potassium-transporting ATPase subunit alpha-1 OS=Homo sapiens OX=9606 GN=ATP1A1 PE=1 SV=1 | | 2 | 2 | | 2 | 2 | | 1023 | | 112.8 | | 5.49 | |  |  |  |
| Q13813 | SPTAN1 | | Spectrin alpha chain, non-erythrocytic 1 OS=Homo sapiens OX=9606 GN=SPTAN1 PE=1 SV=3 | | 11 | 27 | | 29 | 27 | | 2472 | | 284.4 | | 5.35 | |  |  |  |
| Q01082 | SPTBN1 | | Spectrin beta chain, non-erythrocytic 1 OS=Homo sapiens OX=9606 GN=SPTBN1 PE=1 SV=2 | | 7 | 17 | | 19 | 17 | | 2364 | | 274.4 | | 5.57 | |  |  |  |
| Q5JUX0 | SPIN3 | | Spindlin-3 OS=Homo sapiens OX=9606 GN=SPIN3 PE=1 SV=1 | | 4 | 1 | | 1 | 1 | | 258 | | 29.2 | | 6.8 | |  |  |  |
| P23246 | SFPQ | | Splicing factor, proline- and glutamine-rich OS=Homo sapiens OX=9606 GN=SFPQ PE=1 SV=2 | | 3 | 2 | | 2 | 2 | | 707 | | 76.1 | | 9.44 | |  |  |  |
| Q14247 | CTTN | | Src substrate cortactin OS=Homo sapiens OX=9606 GN=CTTN PE=1 SV=2 | | 2 | 1 | | 1 | 1 | | 550 | | 61.5 | | 5.4 | |  |  |  |
| Q93045 | STMN2 | | Stathmin-2 OS=Homo sapiens OX=9606 GN=STMN2 PE=1 SV=3 | | 6 | 1 | | 1 | 1 | | 179 | | 20.8 | | 8.32 | |  |  |  |
| P38646 | HSPA9 | | Stress-70 protein, mitochondrial OS=Homo sapiens OX=9606 GN=HSPA9 PE=1 SV=2 | | 4 | 2 | | 2 | 2 | | 679 | | 73.6 | | 6.16 | |  |  |  |
| Q9NTJ3 | SMC4 | | Structural maintenance of chromosomes protein 4 OS=Homo sapiens OX=9606 GN=SMC4 PE=1 SV=2 | | 2 | 2 | | 2 | 2 | | 1288 | | 147.1 | | 6.79 | |  |  |  |
| P55809 | OXCT1 | | Succinyl-CoA:3-ketoacid coenzyme A transferase 1, mitochondrial OS=Homo sapiens OX=9606 GN=OXCT1 PE=1 SV=1 | | 4 | 2 | | 2 | 2 | | 520 | | 56.1 | | 7.46 | |  |  |  |
| P17987 | TCP1 | | T-complex protein 1 subunit alpha OS=Homo sapiens OX=9606 GN=TCP1 PE=1 SV=1 | | 4 | 2 | | 2 | 2 | | 556 | | 60.3 | | 6.11 | |  |  |  |
| P48643 | CCT5 | | T-complex protein 1 subunit epsilon OS=Homo sapiens OX=9606 GN=CCT5 PE=1 SV=1 | | 1 | 1 | | 1 | 1 | | 541 | | 59.6 | | 5.66 | |  |  |  |
| Q99832 | CCT7 | | T-complex protein 1 subunit eta OS=Homo sapiens OX=9606 GN=CCT7 PE=1 SV=2 | | 2 | 1 | | 1 | 1 | | 543 | | 59.3 | | 7.65 | |  |  |  |
| P50990 | CCT8 | | T-complex protein 1 subunit theta OS=Homo sapiens OX=9606 GN=CCT8 PE=1 SV=4 | | 2 | 1 | | 1 | 1 | | 548 | | 59.6 | | 5.6 | |  |  |  |
| Q15750 | TAB1 | | TGF-beta-activated kinase 1 and MAP3K7-binding protein 1 OS=Homo sapiens OX=9606 GN=TAB1 PE=1 SV=1 | | 4 | 2 | | 2 | 2 | | 504 | | 54.6 | | 5.52 | |  |  |  |
| P26639 | TARS | | Threonine--tRNA ligase, cytoplasmic OS=Homo sapiens OX=9606 GN=TARS PE=1 SV=3 | | 3 | 2 | | 2 | 2 | | 723 | | 83.4 | | 6.67 | |  |  |  |
| P07996 | THBS1 | | Thrombospondin-1 OS=Homo sapiens OX=9606 GN=THBS1 PE=1 SV=2 | | 1 | 1 | | 1 | 1 | | 1170 | | 129.3 | | 4.94 | |  |  |  |
| Q9Y2W1 | THRAP3 | | Thyroid hormone receptor-associated protein 3 OS=Homo sapiens OX=9606 GN=THRAP3 PE=1 SV=2 | | 8 | 7 | | 7 | 7 | | 955 | | 108.6 | | 10.15 | |  |  |  |
| P02786 | TFRC | | Transferrin receptor protein 1 OS=Homo sapiens OX=9606 GN=TFRC PE=1 SV=2 | | 3 | 2 | | 2 | 2 | | 760 | | 84.8 | | 6.61 | |  |  |  |
| P62995 | TRA2B | | Transformer-2 protein homolog beta OS=Homo sapiens OX=9606 GN=TRA2B PE=1 SV=1 | | 11 | 3 | | 3 | 3 | | 288 | | 33.6 | | 11.25 | |  |  |  |
| P37802 | TAGLN2 | | Transgelin-2 OS=Homo sapiens OX=9606 GN=TAGLN2 PE=1 SV=3 | | 4 | 1 | | 1 | 1 | | 199 | | 22.4 | | 8.25 | |  |  |  |
| P55072 | VCP | | Transitional endoplasmic reticulum ATPase OS=Homo sapiens OX=9606 GN=VCP PE=1 SV=4 | | 4 | 3 | | 3 | 3 | | 806 | | 89.3 | | 5.26 | |  |  |  |
| P40939 | HADHA | | Trifunctional enzyme subunit alpha, mitochondrial OS=Homo sapiens OX=9606 GN=HADHA PE=1 SV=2 | | 2 | 1 | | 1 | 1 | | 763 | | 82.9 | | 9.04 | |  |  |  |
| P55084 | HADHB | | Trifunctional enzyme subunit beta, mitochondrial OS=Homo sapiens OX=9606 GN=HADHB PE=1 SV=3 | | 2 | 1 | | 1 | 1 | | 474 | | 51.3 | | 9.41 | |  |  |  |
| P60174 | TPI1 | | Triosephosphate isomerase OS=Homo sapiens OX=9606 GN=TPI1 PE=1 SV=3 | | 4 | 1 | | 1 | 1 | | 286 | | 30.8 | | 5.92 | |  |  |  |
| Q9Y3I0 | RTCB | | tRNA-splicing ligase RtcB homolog OS=Homo sapiens OX=9606 GN=RTCB PE=1 SV=1 | | 2 | 1 | | 1 | 1 | | 505 | | 55.2 | | 7.23 | |  |  |  |
| P68363 | TUBA1B | | Tubulin alpha-1B chain OS=Homo sapiens OX=9606 GN=TUBA1B PE=1 SV=1 | | 4 | 3 | | 4 | 3 | | 451 | | 50.1 | | 5.06 | |  |  |  |
| P68371 | TUBB4B | | Tubulin beta-4B chain OS=Homo sapiens OX=9606 GN=TUBB4B PE=1 SV=1 | | 12 | 5 | | 14 | 5 | | 445 | | 49.8 | | 4.89 | |  |  |  |
| Q9Y4E8 | USP15 | | Ubiquitin carboxyl-terminal hydrolase 15 OS=Homo sapiens OX=9606 GN=USP15 PE=1 SV=3 | | 1 | 1 | | 1 | 1 | | 981 | | 112.3 | | 5.22 | |  |  |  |
| P45974 | USP5 | | Ubiquitin carboxyl-terminal hydrolase 5 OS=Homo sapiens OX=9606 GN=USP5 PE=1 SV=2 | | 1 | 1 | | 1 | 1 | | 858 | | 95.7 | | 5.03 | |  |  |  |
| Q96FW1 | OTUB1 | | Ubiquitin thioesterase OTUB1 OS=Homo sapiens OX=9606 GN=OTUB1 PE=1 SV=2 | | 4 | 1 | | 1 | 1 | | 271 | | 31.3 | | 4.94 | |  |  |  |
| P62979 | RPS27A | | Ubiquitin-40S ribosomal protein S27a OS=Homo sapiens OX=9606 GN=RPS27A PE=1 SV=2 | | 8 | 1 | | 3 | 1 | | 156 | | 18 | | 9.64 | |  |  |  |
| O00159 | MYO1C | | Unconventional myosin-Ic OS=Homo sapiens OX=9606 GN=MYO1C PE=1 SV=4 | | 3 | 3 | | 3 | 3 | | 1063 | | 121.6 | | 9.41 | |  |  |  |
| P08670 | VIM | | Vimentin OS=Homo sapiens OX=9606 GN=VIM PE=1 SV=4 | | 4 | 2 | | 2 | 1 | | 466 | | 53.6 | | 5.12 | |  |  |  |
| P13010 | XRCC5 | | X-ray repair cross-complementing protein 5 OS=Homo sapiens OX=9606 GN=XRCC5 PE=1 SV=3 | | 2 | 1 | | 1 | 1 | | 732 | | 82.7 | | 5.81 | |  |  |  |
| P16989 | YBX3 | | Y-box-binding protein 3 OS=Homo sapiens OX=9606 GN=YBX3 PE=1 SV=4 | | 7 | 2 | | 2 | 1 | | 372 | | 40.1 | | 9.77 | |  |  |  |
| O95218 | ZRANB2 | | Zinc finger Ran-binding domain-containing protein 2 OS=Homo sapiens OX=9606 GN=ZRANB2 PE=1 SV=2 | | 3 | 1 | | 1 | 1 | | 330 | | 37.4 | | 10.01 | |  |  |  |
| Q15942 | ZYX | | Zyxin OS=Homo sapiens OX=9606 GN=ZYX PE=1 SV=1 | | 2 | 1 | | 1 | 1 | | 572 | | 61.2 | | 6.67 | |  |  |  |

| **Table S3. BRINP3- interacting proteins by mass spectrometry (Differential proteins)** | | | | |  |  |  |  |  |
| --- | --- | --- | --- | --- | --- | --- | --- | --- | --- |
| Accession | Gene Name | Description | Coverage [%] | # Peptides | # PSMs | # Unique Peptides | # AAs | MW [kDa] | calc. pI |
| Q76B58 | BRINP3 | BMP/retinoic acid-inducible neural-specific protein 3 OS=Homo sapiens OX=9606 GN=BRINP3 PE=1 SV=1 | 7 | 6 | 6 | 6 | 766 | 88.4 | 7.81 |
| P31946 | YWHAB | 14-3-3 protein beta/alpha OS=Homo sapiens OX=9606 GN=YWHAB PE=1 SV=3 | 17 | 4 | 5 | 2 | 246 | 28.1 | 4.83 |
| P62258 | YWHAE | 14-3-3 protein epsilon OS=Homo sapiens OX=9606 GN=YWHAE PE=1 SV=1 | 12 | 3 | 3 | 2 | 255 | 29.2 | 4.74 |
| P31947 | SFN | 14-3-3 protein sigma OS=Homo sapiens OX=9606 GN=SFN PE=1 SV=1 | 12 | 3 | 3 | 1 | 248 | 27.8 | 4.74 |
| P35998 | PSMC2 | 26S proteasome regulatory subunit 7 OS=Homo sapiens OX=9606 GN=PSMC2 PE=1 SV=3 | 3 | 1 | 1 | 1 | 433 | 48.6 | 5.95 |
| P62244 | RPS15A | 40S ribosomal protein S15a OS=Homo sapiens OX=9606 GN=RPS15A PE=1 SV=2 | 7 | 1 | 1 | 1 | 130 | 14.8 | 10.13 |
| P39019 | RPS19 | 40S ribosomal protein S19 OS=Homo sapiens OX=9606 GN=RPS19 PE=1 SV=2 | 6 | 1 | 1 | 1 | 145 | 16.1 | 10.32 |
| P15880 | RPS2 | 40S ribosomal protein S2 OS=Homo sapiens OX=9606 GN=RPS2 PE=1 SV=2 | 3 | 1 | 1 | 1 | 293 | 31.3 | 10.24 |
| P62851 | RPS25 | 40S ribosomal protein S25 OS=Homo sapiens OX=9606 GN=RPS25 PE=1 SV=1 | 14 | 2 | 2 | 2 | 125 | 13.7 | 10.11 |
| P62701 | RPS4X | 40S ribosomal protein S4, X isoform OS=Homo sapiens OX=9606 GN=RPS4X PE=1 SV=2 | 3 | 1 | 1 | 1 | 263 | 29.6 | 10.15 |
| P46781 | RPS9 | 40S ribosomal protein S9 OS=Homo sapiens OX=9606 GN=RPS9 PE=1 SV=3 | 4 | 1 | 1 | 1 | 194 | 22.6 | 10.65 |
| P10809 | HSPD1 | 60 kDa heat shock protein, mitochondrial OS=Homo sapiens OX=9606 GN=HSPD1 PE=1 SV=2 | 2 | 1 | 1 | 1 | 573 | 61 | 5.87 |
| P05388 | RPLP0 | 60S acidic ribosomal protein P0 OS=Homo sapiens OX=9606 GN=RPLP0 PE=1 SV=1 | 2 | 1 | 1 | 1 | 317 | 34.3 | 5.97 |
| P62906 | RPL10A | 60S ribosomal protein L10a OS=Homo sapiens OX=9606 GN=RPL10A PE=1 SV=2 | 7 | 2 | 2 | 2 | 217 | 24.8 | 9.94 |
| P40429 | RPL13A | 60S ribosomal protein L13a OS=Homo sapiens OX=9606 GN=RPL13A PE=1 SV=2 | 4 | 1 | 1 | 1 | 203 | 23.6 | 10.93 |
| P50914 | RPL14 | 60S ribosomal protein L14 OS=Homo sapiens OX=9606 GN=RPL14 PE=1 SV=4 | 5 | 1 | 1 | 1 | 215 | 23.4 | 10.93 |
| P84098 | RPL19 | 60S ribosomal protein L19 OS=Homo sapiens OX=9606 GN=RPL19 PE=1 SV=1 | 5 | 1 | 1 | 1 | 196 | 23.5 | 11.47 |
| P62750 | RPL23A | 60S ribosomal protein L23a OS=Homo sapiens OX=9606 GN=RPL23A PE=1 SV=1 | 8 | 1 | 1 | 1 | 156 | 17.7 | 10.45 |
| P39023 | RPL3 | 60S ribosomal protein L3 OS=Homo sapiens OX=9606 GN=RPL3 PE=1 SV=2 | 2 | 1 | 1 | 1 | 403 | 46.1 | 10.18 |
| P46777 | RPL5 | 60S ribosomal protein L5 OS=Homo sapiens OX=9606 GN=RPL5 PE=1 SV=3 | 3 | 1 | 1 | 1 | 297 | 34.3 | 9.72 |
| Q02878 | RPL6 | 60S ribosomal protein L6 OS=Homo sapiens OX=9606 GN=RPL6 PE=1 SV=3 | 2 | 1 | 1 | 1 | 288 | 32.7 | 10.58 |
| P62917 | RPL8 | 60S ribosomal protein L8 OS=Homo sapiens OX=9606 GN=RPL8 PE=1 SV=2 | 4 | 1 | 1 | 1 | 257 | 28 | 11.03 |
| P52209 | PGD | 6-phosphogluconate dehydrogenase, decarboxylating OS=Homo sapiens OX=9606 GN=PGD PE=1 SV=3 | 4 | 2 | 2 | 2 | 483 | 53.1 | 7.23 |
| P36639 | NUDT1 | 7,8-dihydro-8-oxoguanine triphosphatase OS=Homo sapiens OX=9606 GN=NUDT1 PE=1 SV=3 | 6 | 1 | 1 | 1 | 197 | 22.5 | 5.27 |
| O15143 | ARPC1B | Actin-related protein 2/3 complex subunit 1B OS=Homo sapiens OX=9606 GN=ARPC1B PE=1 SV=3 | 3 | 1 | 1 | 1 | 372 | 40.9 | 8.35 |
| P30566 | ADSL | Adenylosuccinate lyase OS=Homo sapiens OX=9606 GN=ADSL PE=1 SV=2 | 5 | 2 | 2 | 2 | 484 | 54.9 | 7.11 |
| P02765 | AHSG | Alpha-2-HS-glycoprotein OS=Homo sapiens OX=9606 GN=AHSG PE=1 SV=1 | 3 | 1 | 1 | 1 | 367 | 39.3 | 5.72 |
| O43707 | ACTN4 | Alpha-actinin-4 OS=Homo sapiens OX=9606 GN=ACTN4 PE=1 SV=2 | 1 | 1 | 1 | 1 | 911 | 104.8 | 5.44 |
| P54136 | RARS | Arginine--tRNA ligase, cytoplasmic OS=Homo sapiens OX=9606 GN=RARS PE=1 SV=2 | 1 | 1 | 1 | 1 | 660 | 75.3 | 6.68 |
| P14868 | DARS | Aspartate--tRNA ligase, cytoplasmic OS=Homo sapiens OX=9606 GN=DARS PE=1 SV=2 | 1 | 1 | 1 | 1 | 501 | 57.1 | 6.55 |
| P53396 | ACLY | ATP-citrate synthase OS=Homo sapiens OX=9606 GN=ACLY PE=1 SV=3 | 3 | 3 | 3 | 3 | 1101 | 120.8 | 7.33 |
| O00571 | DDX3X | ATP-dependent RNA helicase DDX3X OS=Homo sapiens OX=9606 GN=DDX3X PE=1 SV=3 | 3 | 2 | 2 | 2 | 662 | 73.2 | 7.18 |
| O95816 | BAG2 | BAG family molecular chaperone regulator 2 OS=Homo sapiens OX=9606 GN=BAG2 PE=1 SV=1 | 9 | 2 | 2 | 2 | 211 | 23.8 | 6.7 |
| O43491 | EPB41L2 | Band 4.1-like protein 2 OS=Homo sapiens OX=9606 GN=EPB41L2 PE=1 SV=1 | 1 | 1 | 1 | 1 | 1005 | 112.5 | 5.44 |
| P07814 | EPRS | Bifunctional glutamate/proline--tRNA ligase OS=Homo sapiens OX=9606 GN=EPRS PE=1 SV=5 | 1 | 2 | 2 | 2 | 1512 | 170.5 | 7.33 |
| P54132 | BLM | Bloom syndrome protein OS=Homo sapiens OX=9606 GN=BLM PE=1 SV=1 | 1 | 1 | 1 | 1 | 1417 | 158.9 | 7.49 |
| Q13557 | CAMK2D | Calcium/calmodulin-dependent protein kinase type II subunit delta OS=Homo sapiens OX=9606 GN=CAMK2D PE=1 SV=3 | 3 | 1 | 1 | 1 | 499 | 56.3 | 7.25 |
| Q9UJS0 | SLC25A13 | Calcium-binding mitochondrial carrier protein Aralar2 OS=Homo sapiens OX=9606 GN=SLC25A13 PE=1 SV=2 | 1 | 1 | 1 | 1 | 675 | 74.1 | 8.62 |
| P27824 | CANX | Calnexin OS=Homo sapiens OX=9606 GN=CANX PE=1 SV=2 | 2 | 1 | 1 | 1 | 592 | 67.5 | 4.6 |
| P07384 | CAPN1 | Calpain-1 catalytic subunit OS=Homo sapiens OX=9606 GN=CAPN1 PE=1 SV=1 | 1 | 1 | 1 | 1 | 714 | 81.8 | 5.67 |
| O43852 | CALU | Calumenin OS=Homo sapiens OX=9606 GN=CALU PE=1 SV=2 | 2 | 1 | 1 | 1 | 315 | 37.1 | 4.64 |
| Q8N163 | CCAR2 | Cell cycle and apoptosis regulator protein 2 OS=Homo sapiens OX=9606 GN=CCAR2 PE=1 SV=2 | 1 | 1 | 1 | 1 | 923 | 102.8 | 5.22 |
| O00299 | CLIC1 | Chloride intracellular channel protein 1 OS=Homo sapiens OX=9606 GN=CLIC1 PE=1 SV=4 | 8 | 2 | 2 | 2 | 241 | 26.9 | 5.17 |
| Q9Y696 | CLIC4 | Chloride intracellular channel protein 4 OS=Homo sapiens OX=9606 GN=CLIC4 PE=1 SV=4 | 8 | 2 | 2 | 2 | 253 | 28.8 | 5.59 |
| P09496 | CLTA | Clathrin light chain A OS=Homo sapiens OX=9606 GN=CLTA PE=1 SV=1 | 4 | 1 | 1 | 1 | 248 | 27.1 | 4.51 |
| P06493 | CDK1 | Cyclin-dependent kinase 1 OS=Homo sapiens OX=9606 GN=CDK1 PE=1 SV=3 | 3 | 1 | 1 | 1 | 297 | 34.1 | 8.4 |
| P22695 | UQCRC2 | Cytochrome b-c1 complex subunit 2, mitochondrial OS=Homo sapiens OX=9606 GN=UQCRC2 PE=1 SV=3 | 4 | 1 | 1 | 1 | 453 | 48.4 | 8.63 |
| P21399 | ACO1 | Cytoplasmic aconitate hydratase OS=Homo sapiens OX=9606 GN=ACO1 PE=1 SV=3 | 1 | 1 | 1 | 1 | 889 | 98.3 | 6.68 |
| Q96KP4 | CNDP2 | Cytosolic non-specific dipeptidase OS=Homo sapiens OX=9606 GN=CNDP2 PE=1 SV=2 | 2 | 1 | 1 | 1 | 475 | 52.8 | 5.97 |
| Q13268 | DHRS2 | Dehydrogenase/reductase SDR family member 2, mitochondrial OS=Homo sapiens OX=9606 GN=DHRS2 PE=1 SV=4 | 4 | 1 | 1 | 1 | 280 | 29.9 | 9.01 |
| P09622 | DLD | Dihydrolipoyl dehydrogenase, mitochondrial OS=Homo sapiens OX=9606 GN=DLD PE=1 SV=2 | 2 | 1 | 1 | 1 | 509 | 54.1 | 7.85 |
| P49736 | MCM2 | DNA replication licensing factor MCM2 OS=Homo sapiens OX=9606 GN=MCM2 PE=1 SV=4 | 1 | 1 | 1 | 1 | 904 | 101.8 | 5.52 |
| P33993 | MCM7 | DNA replication licensing factor MCM7 OS=Homo sapiens OX=9606 GN=MCM7 PE=1 SV=4 | 1 | 1 | 1 | 1 | 719 | 81.3 | 6.46 |
| Q14258 | TRIM25 | E3 ubiquitin/ISG15 ligase TRIM25 OS=Homo sapiens OX=9606 GN=TRIM25 PE=1 SV=2 | 2 | 1 | 1 | 1 | 630 | 70.9 | 8.09 |
| P13639 | EEF2 | Elongation factor 2 OS=Homo sapiens OX=9606 GN=EEF2 PE=1 SV=4 | 3 | 3 | 3 | 3 | 858 | 95.3 | 6.83 |
| P14625 | HSP90B1 | Endoplasmin OS=Homo sapiens OX=9606 GN=HSP90B1 PE=1 SV=1 | 5 | 5 | 5 | 4 | 803 | 92.4 | 4.84 |
| O75477 | ERLIN1 | Erlin-1 OS=Homo sapiens OX=9606 GN=ERLIN1 PE=1 SV=1 | 4 | 1 | 1 | 1 | 346 | 38.9 | 7.87 |
| P05198 | EIF2S1 | Eukaryotic translation initiation factor 2 subunit 1 OS=Homo sapiens OX=9606 GN=EIF2S1 PE=1 SV=3 | 5 | 2 | 2 | 2 | 315 | 36.1 | 5.08 |
| P41091 | EIF2S3 | Eukaryotic translation initiation factor 2 subunit 3 OS=Homo sapiens OX=9606 GN=EIF2S3 PE=1 SV=3 | 2 | 1 | 1 | 1 | 472 | 51.1 | 8.4 |
| Q14152 | EIF3A | Eukaryotic translation initiation factor 3 subunit A OS=Homo sapiens OX=9606 GN=EIF3A PE=1 SV=1 | 1 | 1 | 1 | 1 | 1382 | 166.5 | 6.79 |
| P55884 | EIF3B | Eukaryotic translation initiation factor 3 subunit B OS=Homo sapiens OX=9606 GN=EIF3B PE=1 SV=3 | 2 | 1 | 1 | 1 | 814 | 92.4 | 5 |
| B5ME19 | EIF3CL | Eukaryotic translation initiation factor 3 subunit C-like protein OS=Homo sapiens OX=9606 GN=EIF3CL PE=3 SV=1 | 1 | 1 | 1 | 1 | 914 | 105.4 | 5.64 |
| O00303 | EIF3F | Eukaryotic translation initiation factor 3 subunit F OS=Homo sapiens OX=9606 GN=EIF3F PE=1 SV=1 | 3 | 1 | 1 | 1 | 357 | 37.5 | 5.45 |
| P78344 | EIF4G2 | Eukaryotic translation initiation factor 4 gamma 2 OS=Homo sapiens OX=9606 GN=EIF4G2 PE=1 SV=1 | 2 | 2 | 2 | 2 | 907 | 102.3 | 7.14 |
| P15311 | EZR | Ezrin OS=Homo sapiens OX=9606 GN=EZR PE=1 SV=4 | 1 | 1 | 1 | 1 | 586 | 69.4 | 6.27 |
| Q9Y5B9 | SUPT16H | FACT complex subunit SPT16 OS=Homo sapiens OX=9606 GN=SUPT16H PE=1 SV=1 | 1 | 1 | 1 | 1 | 1047 | 119.8 | 5.66 |
| P52907 | CAPZA1 | F-actin-capping protein subunit alpha-1 OS=Homo sapiens OX=9606 GN=CAPZA1 PE=1 SV=3 | 5 | 1 | 1 | 1 | 286 | 32.9 | 5.69 |
| Q96AC1 | FERMT2 | Fermitin family homolog 2 OS=Homo sapiens OX=9606 GN=FERMT2 PE=1 SV=1 | 2 | 1 | 1 | 1 | 680 | 77.8 | 6.7 |
| P04075 | ALDOA | Fructose-bisphosphate aldolase A OS=Homo sapiens OX=9606 GN=ALDOA PE=1 SV=2 | 5 | 2 | 2 | 2 | 364 | 39.4 | 8.09 |
| P06396 | GSN | Gelsolin OS=Homo sapiens OX=9606 GN=GSN PE=1 SV=1 | 2 | 2 | 2 | 2 | 782 | 85.6 | 6.28 |
| P06744 | GPI | Glucose-6-phosphate isomerase OS=Homo sapiens OX=9606 GN=GPI PE=1 SV=4 | 2 | 1 | 1 | 1 | 558 | 63.1 | 8.32 |
| P0DMV8 | HSPA1A | Heat shock 70 kDa protein 1A OS=Homo sapiens OX=9606 GN=HSPA1A PE=1 SV=1 | 5 | 4 | 4 | 1 | 641 | 70 | 5.66 |
| P52272 | HNRNPM | Heterogeneous nuclear ribonucleoprotein M OS=Homo sapiens OX=9606 GN=HNRNPM PE=1 SV=3 | 2 | 2 | 2 | 2 | 730 | 77.5 | 8.7 |
| P07910 | HNRNPC | Heterogeneous nuclear ribonucleoproteins C1/C2 OS=Homo sapiens OX=9606 GN=HNRNPC PE=1 SV=4 | 3 | 1 | 1 | 1 | 306 | 33.7 | 5.08 |
| O00422 | SAP18 | Histone deacetylase complex subunit SAP18 OS=Homo sapiens OX=9606 GN=SAP18 PE=1 SV=1 | 5 | 1 | 1 | 1 | 153 | 17.6 | 9.35 |
| P10412 | HIST1H1E | Histone H1.4 OS=Homo sapiens OX=9606 GN=HIST1H1E PE=1 SV=2 | 22 | 5 | 6 | 1 | 219 | 21.9 | 11.03 |
| P16104 | H2AFX | Histone H2AX OS=Homo sapiens OX=9606 GN=H2AFX PE=1 SV=2 | 6 | 1 | 1 | 1 | 143 | 15.1 | 10.74 |
| O60814 | HIST1H2BK | Histone H2B type 1-K OS=Homo sapiens OX=9606 GN=HIST1H2BK PE=1 SV=3 | 14 | 2 | 2 | 2 | 126 | 13.9 | 10.32 |
| Q86YZ3 | HRNR | Hornerin OS=Homo sapiens OX=9606 GN=HRNR PE=1 SV=2 | 3 | 1 | 1 | 1 | 2850 | 282.2 | 10.04 |
| Q01581 | HMGCS1 | Hydroxymethylglutaryl-CoA synthase, cytoplasmic OS=Homo sapiens OX=9606 GN=HMGCS1 PE=1 SV=2 | 3 | 1 | 1 | 1 | 520 | 57.3 | 5.41 |
| P0DOX5 |  | Immunoglobulin gamma-1 heavy chain OS=Homo sapiens OX=9606 PE=1 SV=2 | 2 | 1 | 1 | 1 | 449 | 49.3 | 8.72 |
| Q13418 | ILK | Integrin-linked protein kinase OS=Homo sapiens OX=9606 GN=ILK PE=1 SV=2 | 2 | 1 | 1 | 1 | 452 | 51.4 | 8.07 |
| Q12906 | ILF3 | Interleukin enhancer-binding factor 3 OS=Homo sapiens OX=9606 GN=ILF3 PE=1 SV=3 | 1 | 1 | 1 | 1 | 894 | 95.3 | 8.76 |
| P20700 | LMNB1 | Lamin-B1 OS=Homo sapiens OX=9606 GN=LMNB1 PE=1 SV=2 | 5 | 3 | 3 | 2 | 586 | 66.4 | 5.16 |
| P42704 | LRPPRC | Leucine-rich PPR motif-containing protein, mitochondrial OS=Homo sapiens OX=9606 GN=LRPPRC PE=1 SV=3 | 1 | 1 | 1 | 1 | 1394 | 157.8 | 6.13 |
| Q9P2J5 | LARS | Leucine--tRNA ligase, cytoplasmic OS=Homo sapiens OX=9606 GN=LARS PE=1 SV=2 | 1 | 1 | 1 | 1 | 1176 | 134.4 | 7.3 |
| P09960 | LTA4H | Leukotriene A-4 hydrolase OS=Homo sapiens OX=9606 GN=LTA4H PE=1 SV=2 | 2 | 1 | 1 | 1 | 611 | 69.2 | 6.18 |
| Q9UHB6 | LIMA1 | LIM domain and actin-binding protein 1 OS=Homo sapiens OX=9606 GN=LIMA1 PE=1 SV=1 | 1 | 1 | 1 | 1 | 759 | 85.2 | 6.84 |
| P43361 | MAGEA8 | Melanoma-associated antigen 8 OS=Homo sapiens OX=9606 GN=MAGEA8 PE=1 SV=2 | 5 | 2 | 2 | 2 | 318 | 35.2 | 4.77 |
| Q9UNF1 | MAGED2 | Melanoma-associated antigen D2 OS=Homo sapiens OX=9606 GN=MAGED2 PE=1 SV=2 | 3 | 2 | 2 | 2 | 606 | 64.9 | 9.32 |
| P56192 | MARS | Methionine--tRNA ligase, cytoplasmic OS=Homo sapiens OX=9606 GN=MARS PE=1 SV=2 | 1 | 1 | 1 | 1 | 900 | 101.1 | 6.16 |
| P27816 | MAP4 | Microtubule-associated protein 4 OS=Homo sapiens OX=9606 GN=MAP4 PE=1 SV=3 | 3 | 2 | 2 | 2 | 1152 | 120.9 | 5.43 |
| Q6P1R3 | MSANTD2 | Myb/SANT-like DNA-binding domain-containing protein 2 OS=Homo sapiens OX=9606 GN=MSANTD2 PE=1 SV=1 | 2 | 1 | 1 | 1 | 559 | 61.3 | 6.19 |
| Q13423 | NNT | NAD(P) transhydrogenase, mitochondrial OS=Homo sapiens OX=9606 GN=NNT PE=1 SV=3 | 1 | 1 | 1 | 1 | 1086 | 113.8 | 8.09 |
| P16435 | POR | NADPH--cytochrome P450 reductase OS=Homo sapiens OX=9606 GN=POR PE=1 SV=2 | 1 | 1 | 1 | 1 | 677 | 76.6 | 5.58 |
| Q09666 | AHNAK | Neuroblast differentiation-associated protein AHNAK OS=Homo sapiens OX=9606 GN=AHNAK PE=1 SV=2 | 2 | 3 | 4 | 3 | 5890 | 628.7 | 6.15 |
| Q0ZGT2 | NEXN | Nexilin OS=Homo sapiens OX=9606 GN=NEXN PE=1 SV=1 | 2 | 1 | 1 | 1 | 675 | 80.6 | 5.33 |
| P43490 | NAMPT | Nicotinamide phosphoribosyltransferase OS=Homo sapiens OX=9606 GN=NAMPT PE=1 SV=1 | 1 | 1 | 1 | 1 | 491 | 55.5 | 7.15 |
| P49321 | NASP | Nuclear autoantigenic sperm protein OS=Homo sapiens OX=9606 GN=NASP PE=1 SV=2 | 1 | 1 | 1 | 1 | 788 | 85.2 | 4.3 |
| Q9NR30 | DDX21 | Nucleolar RNA helicase 2 OS=Homo sapiens OX=9606 GN=DDX21 PE=1 SV=5 | 2 | 1 | 1 | 1 | 783 | 87.3 | 9.28 |
| P06748 | NPM1 | Nucleophosmin OS=Homo sapiens OX=9606 GN=NPM1 PE=1 SV=2 | 10 | 3 | 4 | 3 | 294 | 32.6 | 4.78 |
| Q9Y5B8 | NME7 | Nucleoside diphosphate kinase 7 OS=Homo sapiens OX=9606 GN=NME7 PE=1 SV=1 | 3 | 1 | 1 | 1 | 376 | 42.5 | 6.47 |
| Q8WX93 | PALLD | Palladin OS=Homo sapiens OX=9606 GN=PALLD PE=1 SV=3 | 1 | 1 | 1 | 1 | 1383 | 150.5 | 7.09 |
| Q06830 | PRDX1 | Peroxiredoxin-1 OS=Homo sapiens OX=9606 GN=PRDX1 PE=1 SV=1 | 14 | 3 | 3 | 3 | 199 | 22.1 | 8.13 |
| O00541 | PES1 | Pescadillo homolog OS=Homo sapiens OX=9606 GN=PES1 PE=1 SV=1 | 1 | 1 | 1 | 1 | 588 | 68 | 7.33 |
| Q9Y263 | PLAA | Phospholipase A-2-activating protein OS=Homo sapiens OX=9606 GN=PLAA PE=1 SV=2 | 2 | 1 | 1 | 1 | 795 | 87.1 | 6.37 |
| Q9Y617 | PSAT1 | Phosphoserine aminotransferase OS=Homo sapiens OX=9606 GN=PSAT1 PE=1 SV=2 | 3 | 1 | 1 | 1 | 370 | 40.4 | 7.66 |
| P09874 | PARP1 | Poly [ADP-ribose] polymerase 1 OS=Homo sapiens OX=9606 GN=PARP1 PE=1 SV=4 | 1 | 1 | 1 | 1 | 1014 | 113 | 8.88 |
| Q15365 | PCBP1 | Poly(rC)-binding protein 1 OS=Homo sapiens OX=9606 GN=PCBP1 PE=1 SV=2 | 8 | 3 | 3 | 3 | 356 | 37.5 | 7.09 |
| P11940 | PABPC1 | Polyadenylate-binding protein 1 OS=Homo sapiens OX=9606 GN=PABPC1 PE=1 SV=2 | 3 | 2 | 2 | 2 | 636 | 70.6 | 9.5 |
| P02545 | LMNA | Prelamin-A/C OS=Homo sapiens OX=9606 GN=LMNA PE=1 SV=1 | 3 | 2 | 2 | 1 | 664 | 74.1 | 7.02 |
| Q92841 | DDX17 | Probable ATP-dependent RNA helicase DDX17 OS=Homo sapiens OX=9606 GN=DDX17 PE=1 SV=2 | 2 | 1 | 1 | 1 | 729 | 80.2 | 8.27 |
| P12004 | PCNA | Proliferating cell nuclear antigen OS=Homo sapiens OX=9606 GN=PCNA PE=1 SV=1 | 5 | 1 | 1 | 1 | 261 | 28.8 | 4.69 |
| Q32P28 | P3H1 | Prolyl 3-hydroxylase 1 OS=Homo sapiens OX=9606 GN=P3H1 PE=1 SV=2 | 3 | 2 | 2 | 2 | 736 | 83.3 | 5.14 |
| P25789 | PSMA4 | Proteasome subunit alpha type-4 OS=Homo sapiens OX=9606 GN=PSMA4 PE=1 SV=1 | 3 | 1 | 1 | 1 | 261 | 29.5 | 7.72 |
| P30101 | PDIA3 | Protein disulfide-isomerase A3 OS=Homo sapiens OX=9606 GN=PDIA3 PE=1 SV=4 | 3 | 1 | 1 | 1 | 505 | 56.7 | 6.35 |
| Q15084 | PDIA6 | Protein disulfide-isomerase A6 OS=Homo sapiens OX=9606 GN=PDIA6 PE=1 SV=1 | 3 | 1 | 1 | 1 | 440 | 48.1 | 5.08 |
| P07237 | P4HB | Protein disulfide-isomerase OS=Homo sapiens OX=9606 GN=P4HB PE=1 SV=3 | 2 | 1 | 1 | 1 | 508 | 57.1 | 4.87 |
| P53992 | SEC24C | Protein transport protein Sec24C OS=Homo sapiens OX=9606 GN=SEC24C PE=1 SV=3 | 1 | 1 | 1 | 1 | 1094 | 118.2 | 7.06 |
| Q96MG8 | PCMTD1 | Protein-L-isoaspartate O-methyltransferase domain-containing protein 1 OS=Homo sapiens OX=9606 GN=PCMTD1 PE=1 SV=2 | 2 | 1 | 1 | 1 | 357 | 40.7 | 5.66 |
| P14618 | PKM | Pyruvate kinase PKM OS=Homo sapiens OX=9606 GN=PKM PE=1 SV=4 | 4 | 2 | 2 | 2 | 531 | 57.9 | 7.84 |
| P63244 | RACK1 | Receptor of activated protein C kinase 1 OS=Homo sapiens OX=9606 GN=RACK1 PE=1 SV=3 | 3 | 1 | 1 | 1 | 317 | 35.1 | 7.69 |
| Q13464 | ROCK1 | Rho-associated protein kinase 1 OS=Homo sapiens OX=9606 GN=ROCK1 PE=1 SV=1 | 1 | 1 | 1 | 1 | 1354 | 158.1 | 5.9 |
| P13489 | RNH1 | Ribonuclease inhibitor OS=Homo sapiens OX=9606 GN=RNH1 PE=1 SV=2 | 2 | 1 | 1 | 1 | 461 | 49.9 | 4.82 |
| P34897 | SHMT2 | Serine hydroxymethyltransferase, mitochondrial OS=Homo sapiens OX=9606 GN=SHMT2 PE=1 SV=3 | 2 | 1 | 1 | 1 | 504 | 56 | 8.53 |
| Q07955 | SRSF1 | Serine/arginine-rich splicing factor 1 OS=Homo sapiens OX=9606 GN=SRSF1 PE=1 SV=2 | 4 | 1 | 1 | 1 | 248 | 27.7 | 10.36 |
| Q9BRL6 | SRSF8 | Serine/arginine-rich splicing factor 8 OS=Homo sapiens OX=9606 GN=SRSF8 PE=1 SV=1 | 5 | 2 | 2 | 2 | 282 | 32.3 | 11.72 |
| P62136 | PPP1CA | Serine/threonine-protein phosphatase PP1-alpha catalytic subunit OS=Homo sapiens OX=9606 GN=PPP1CA PE=1 SV=1 | 3 | 1 | 1 | 1 | 330 | 37.5 | 6.33 |
| Q9BXP5 | SRRT | Serrate RNA effector molecule homolog OS=Homo sapiens OX=9606 GN=SRRT PE=1 SV=1 | 1 | 1 | 1 | 1 | 876 | 100.6 | 5.96 |
| Q9P270 | SLAIN2 | SLAIN motif-containing protein 2 OS=Homo sapiens OX=9606 GN=SLAIN2 PE=1 SV=2 | 3 | 1 | 1 | 1 | 581 | 62.5 | 9.45 |
| P55854 | SUMO3 | Small ubiquitin-related modifier 3 OS=Homo sapiens OX=9606 GN=SUMO3 PE=1 SV=2 | 12 | 1 | 1 | 1 | 103 | 11.6 | 5.49 |
| P05023 | ATP1A1 | Sodium/potassium-transporting ATPase subunit alpha-1 OS=Homo sapiens OX=9606 GN=ATP1A1 PE=1 SV=1 | 2 | 2 | 2 | 2 | 1023 | 112.8 | 5.49 |
| Q14247 | CTTN | Src substrate cortactin OS=Homo sapiens OX=9606 GN=CTTN PE=1 SV=2 | 2 | 1 | 1 | 1 | 550 | 61.5 | 5.4 |
| Q93045 | STMN2 | Stathmin-2 OS=Homo sapiens OX=9606 GN=STMN2 PE=1 SV=3 | 6 | 1 | 1 | 1 | 179 | 20.8 | 8.32 |
| Q9NTJ3 | SMC4 | Structural maintenance of chromosomes protein 4 OS=Homo sapiens OX=9606 GN=SMC4 PE=1 SV=2 | 2 | 2 | 2 | 2 | 1288 | 147.1 | 6.79 |
| P55809 | OXCT1 | Succinyl-CoA:3-ketoacid coenzyme A transferase 1, mitochondrial OS=Homo sapiens OX=9606 GN=OXCT1 PE=1 SV=1 | 4 | 2 | 2 | 2 | 520 | 56.1 | 7.46 |
| P17987 | TCP1 | T-complex protein 1 subunit alpha OS=Homo sapiens OX=9606 GN=TCP1 PE=1 SV=1 | 4 | 2 | 2 | 2 | 556 | 60.3 | 6.11 |
| P50990 | CCT8 | T-complex protein 1 subunit theta OS=Homo sapiens OX=9606 GN=CCT8 PE=1 SV=4 | 2 | 1 | 1 | 1 | 548 | 59.6 | 5.6 |
| P26639 | TARS | Threonine--tRNA ligase, cytoplasmic OS=Homo sapiens OX=9606 GN=TARS PE=1 SV=3 | 3 | 2 | 2 | 2 | 723 | 83.4 | 6.67 |
| P07996 | THBS1 | Thrombospondin-1 OS=Homo sapiens OX=9606 GN=THBS1 PE=1 SV=2 | 1 | 1 | 1 | 1 | 1170 | 129.3 | 4.94 |
| P37802 | TAGLN2 | Transgelin-2 OS=Homo sapiens OX=9606 GN=TAGLN2 PE=1 SV=3 | 4 | 1 | 1 | 1 | 199 | 22.4 | 8.25 |
| P60174 | TPI1 | Triosephosphate isomerase OS=Homo sapiens OX=9606 GN=TPI1 PE=1 SV=3 | 4 | 1 | 1 | 1 | 286 | 30.8 | 5.92 |
| Q9Y3I0 | RTCB | tRNA-splicing ligase RtcB homolog OS=Homo sapiens OX=9606 GN=RTCB PE=1 SV=1 | 2 | 1 | 1 | 1 | 505 | 55.2 | 7.23 |
| P45974 | USP5 | Ubiquitin carboxyl-terminal hydrolase 5 OS=Homo sapiens OX=9606 GN=USP5 PE=1 SV=2 | 1 | 1 | 1 | 1 | 858 | 95.7 | 5.03 |
| Q96FW1 | OTUB1 | Ubiquitin thioesterase OTUB1 OS=Homo sapiens OX=9606 GN=OTUB1 PE=1 SV=2 | 4 | 1 | 1 | 1 | 271 | 31.3 | 4.94 |
| P62979 | RPS27A | Ubiquitin-40S ribosomal protein S27a OS=Homo sapiens OX=9606 GN=RPS27A PE=1 SV=2 | 8 | 1 | 3 | 1 | 156 | 18 | 9.64 |
| P13010 | XRCC5 | X-ray repair cross-complementing protein 5 OS=Homo sapiens OX=9606 GN=XRCC5 PE=1 SV=3 | 2 | 1 | 1 | 1 | 732 | 82.7 | 5.81 |
| O95218 | ZRANB2 | Zinc finger Ran-binding domain-containing protein 2 OS=Homo sapiens OX=9606 GN=ZRANB2 PE=1 SV=2 | 3 | 1 | 1 | 1 | 330 | 37.4 | 10.01 |
| Q15942 | ZYX | Zyxin OS=Homo sapiens OX=9606 GN=ZYX PE=1 SV=1 | 2 | 1 | 1 | 1 | 572 | 61.2 | 6.67 |
